# Exposure age and ice-sheet model constraints on Pliocene East Antarctic ice sheet dynamics

**DOI:** 10.1038/ncomms8016

**Published:** 2015-04-24

**Authors:** Masako Yamane, Yusuke Yokoyama, Ayako Abe-Ouchi, Stephen Obrochta, Fuyuki Saito, Kiichi Moriwaki, Hiroyuki Matsuzaki

**Affiliations:** 1 Atmosphere and Ocean Research Institute, University of Tokyo, 5-1-5 Kashiwanoha, Chiba 277-8564, Japan; 2 Department of Earth and Planetary Sciences, University of Tokyo, 7-3-1 Hongo, Bunkyoku, Tokyo 113-0033, Japan; 3 Japan Agency for Marine-Earth Science and Technology, 2-15 Natsushima-cho, Yokosuka 237-0061, Japan; 4 Japan Agency for Marine-Earth Science and Technology, 3173-25, Showa-machi, Kanazawa-ku, Yokohama, Kanagawa 236-0001, Japan; 5 Faculty of International Resource Science, Akita University 1-1 Tegata Gakuin-cho, Akita 010-8502, Japan; 6 National Institute of Polar Research, 10-3, Midoricho, Tachikawa, Tokyo 190-8518, Japan; 7 The University Museum, University of Tokyo, 7-3-1 Hongo, Bunkyo-ku, Tokyo 113-0033, Japan

## Abstract

The Late Pliocene epoch is a potential analogue for future climate in a warming world. Here we reconstruct Plio-Pleistocene East Antarctic Ice Sheet (EAIS) variability using cosmogenic nuclide exposure ages and model simulations to better understand ice sheet behaviour under such warm conditions. New and previously published exposure ages indicate interior-thickening during the Pliocene. An ice sheet model with mid-Pliocene boundary conditions also results in interior thickening and suggests that both the Wilkes Subglacial and Aurora Basins largely melted, offsetting increased ice volume. Considering contributions from West Antarctica and Greenland, this is consistent with the most recent IPCC AR5 estimate, which indicates that the Pliocene sea level likely did not exceed +20 m on Milankovitch timescales. The inception of colder climate since ∼3 Myr has increased the sea ice cover and inhibited active moisture transport to Antarctica, resulting in reduced ice sheet thickness, at least in coastal areas.

## Abstract (web summary)

The behaviour of Antarctic ice sheets during warm climates of the past is poorly understood. Here, the authors combine cosmogenic nuclide exposure ages and numerical simulations in an effort to assess changes in East Antarctic ice sheet thickness since the Pliocene.

## Graphical abstract

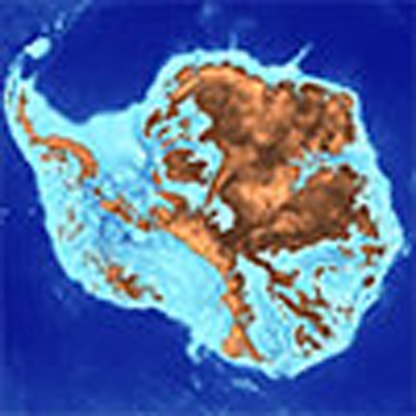

The climatic optimum of the Late Pliocene epoch (3.3–3.0 Myr) is often considered an analogue for near future conditions^1^,^2^ because of globally warmer temperatures and similar CO_2_ concentrations relative to today^3^,^4^,^5^,^6^,^7^,^8^. This resulted in lower continental ice volume, in addition to which there is ‘high confidence' of higher global sea level during the Pliocene^2^. Published reconstructions of the maximum Pliocene sea level vary between+5 and+50 m compared with present^9^,^10^,^11^,^12^,^13^, with the current best estimate being the IPCC AR5 value of no more than +20 m (ref. 2). The Greenland Ice Sheet and the West Antarctic Ice Sheet (WAIS), both of which are thought to have largely melted during Pliocene interglacials^14^,^15^,^16^, currently store ∼13 m sea-level equivalent (SLE) ice. Modelling studies^17^,^18^ and recent observational evidence^19^,^20^ indicate that the margins of East Antarctica, especially the Wilkes Subglacial and Aurora Basins, further contributed to high Pliocene sea levels.

However, there is relatively little evidence directly constraining the response of the East Antarctic Ice Sheet (EAIS) to Pliocene climate evolution. Thick Cenozoic glaciomarine sequences are exposed at Mac Robertson Land (MRL) and the Transantarctic Mountains (Pagodroma Group and Sirius Group, respectively), suggesting that the ice margin was located several hundred kilometres inland of its present location during the Miocene or Pliocene^21^,^22^. An ∼80-m diatomite section recovered in the ANDRILL core indicates higher Pliocene phytoplankton productivity in a largely ice-free Ross Sea^15^. During the Late Pliocene, the occurrence of diamictons in the Prydz Bay (ODP Site 1167 (ref. 23)) suggests glacial expansion.

To better constrain EAIS dynamics during the Pliocene, we present newly acquired and recently compiled *in situ* cosmogenic nuclide exposures ages together with model simulations of Antarctic ice thickness and distribution. We first reconstruct Pliocene EAIS configuration from the cumulative exposure time of bedrock, as reflected by the concentration of *in situ* produced cosmogenic nuclides (for example, ^3^He, ^10^Be, ^14^C, ^21^Ne, ^26^Al and ^36^Cl) in quartz from ice-free regions. The altitude of each sampling sites in turn provides a minimum estimate of ice sheet thickness at that location. We then compare this result with our model-based reconstruction to make inference on the ice sheet history of currently glaciated areas, such as the Wilkes Subglacial and Aurora Basins, from which geologic data are scarce. We finally use the ratio of two cosmogenic nuclides (for example, ^26^Al/^10^Be) to determine exposure history (simple or complex).

## Results

### New and compiled exposure ages

**Figure 1:**
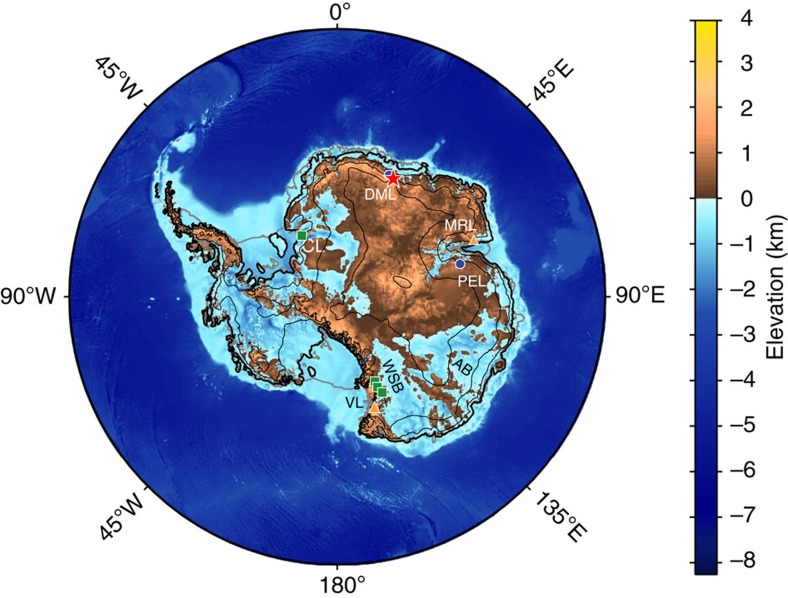
Antarctic bedrock elevation. Antarctic bedrock elevation with grounded ice sheet boundary and attached, floating ice shelves. Thick black line shows grounded ice sheet boundary and thick grey lines show floating ice shelves. Thin black lines show ice sheet elevation (1,000 m contour interval^70^). Location of exposure ages reported here is shown with a red star and all other Area 1 sites with blue circles. Area 2 and 3 sites are shown with green squares and orange triangles, respectively. CL is Coats Land, DML is Droning Maud Land and MRL is Mac. Robertson Land, PEL is Princess Elizabeth Land and VL is Victoria Land. AB and WSB refer to the Aurora Basin and Wilkes Subglacial Basin, respectively.

New ^10^Be and ^26^Al data presented here were obtained from material collected at the central and eastern portions of the Sør Rondane Mountains, Droning Maud Land (DML) by the National Institute for Polar Research during JARE 27, 28, 31 and 32 (Fig. 1; Supplementary Table 1). The Sør Rondane Mountains form the eastern portion of a discontinuous, ∼2,000 km east–west trending range that is located 200–400 km inland^24^ (Supplementary Fig. 1). There are a series of glacial tills located at varying altitudes in the Sør Rondane Mountains. These tills indicate five distinct exposure stages, the oldest (4 Myr) and most weathered of which is located at ∼400 m above the present ice surface^24^. Exposure age and weathering extent of the tills progressively decreases with altitude to a minimum of ∼40 ka at 3 m above the present ice sheet. This indicates that the ice sheet at this location was 400 m thicker than present before 4 Myr and gradually thinned to its current height^24^.

Previously published exposure ages are compiled from five ice-free areas of East Antarctica and grouped on the basis of distance from the ice sheet edge (Fig. 1). Area 1 (Supplementary Note 1) is ∼300 km inland and includes our new data with previously published ages^25^,^26^ from the Sør Rondane Mountains in DML (Supplementary Fig. 1), as well as previously reported data from the Grove Mountains^27^,^28^,^29^,^30^ in Princess Elizabeth Land (Supplementary Fig. 2). Area 2 (Supplementary Note 2) includes exposure ages from samples and outcrops that are located ∼100 km inland and encompasses the Richer Hills^31^ (Supplementary Fig. 3), the Allan Hills^25^,^32^ (Supplementary Fig. 4), the McMurdo Sound–Dry Valleys^33^,^34^,^35^ (Supplementary Fig. 5) in Victoria Land (VL) and the Shackleton Range^36^ in Coats Land (CL; Supplementary Fig. 6). Area 3 (Supplementary Note 3) includes exposure ages from samples and outcrops that are proximal to the ice sheet edge and includes Prince Charles Mountains^37^ in MRL (Supplementary Fig. 7) and the Deep Freeze Range^38^,^39^ (Supplementary Fig. 8) in VL.

### Area 1

**Figure 2:**
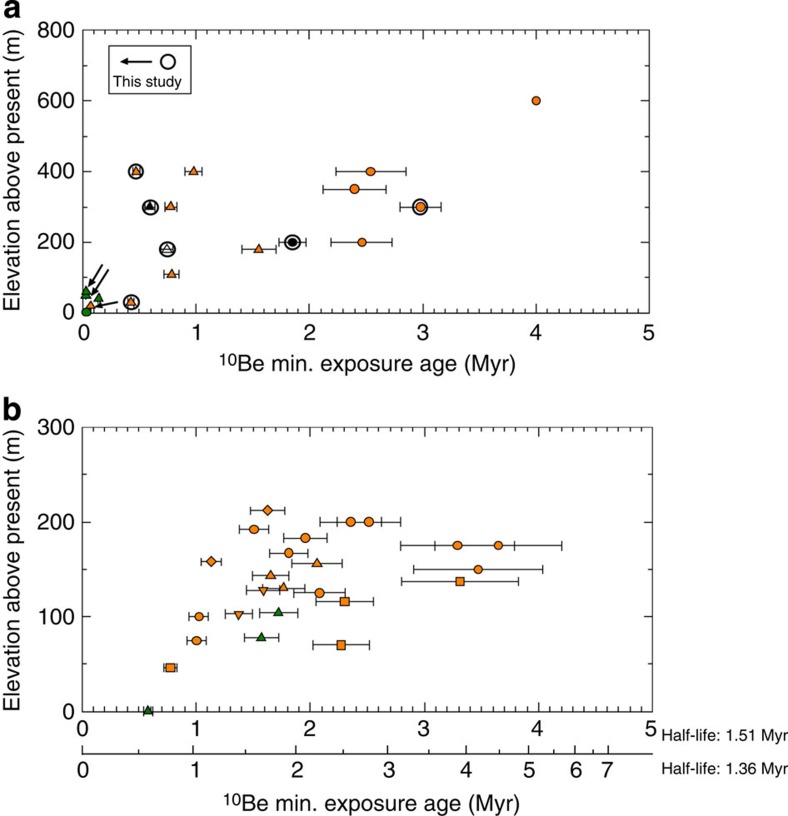
Area 1 ice sheet history. (**a**) Exposure ages from the Sør Rondane Mountains from this study (denoted in Fig. 1) and previous studies^25^,^26^. Circles and triangles indicate samples north (downstream) and south (upstream) of 72 °S, respectively. The black symbol indicates samples lacking ^26^Al measurement, and filled and open symbols refer to bedrock and erratic samples, respectively. (**b**) Compiled Grove Mountains exposure ages^27^,^28^,^29^,^30^. In both panels, 1*σ* uncertainty is shown, and orange and green symbols indicate simple and complex exposure history, respectively.

We report ten ^10^Be and six ^26^Al ages from the Sør Rondane Mountains. Two samples exhibit a complex exposure history (Supplementary Fig. 9; Supplementary Table 2). We also review previously published exposure ages^25^,^26^ from Sør Rondane Mountains, several of which were calculated with standard atmospheric thickness (see Methods). Where appropriate, we revise the ages using the scaling factor of Stone (ref. 40). Our new data together with previously reported ages from the Sør Rondane Mountains indicate that the EAIS in this area was thicker by at least 400 m than present between ∼2.5 and ∼3 Myr (Fig. 2a); however, the presence of an age (near the limit of ^10^Be dating) obtained from a higher altitude raises the possibility that the ice sheet was ∼600 m above present on at least one occasion before 4 Myr. Exposure histories are predominately complex during the Late Pleistocene, with simple exposure histories predominating for samples with larger exposure ages. The subsequent thinning pattern varies between the upstream and downstream portions of the ice sheet. Upstream ice thickness began decreasing during the Pliocene, declining to ∼200 m above present by ∼2.5±0.5 Myr. Downstream ice thickness declined more rapidly, beginning at ∼1.0±0.1 Myr, and reached the current level at ∼0.4 Myr. These results are consistent with geomorphologic evidence from glacial tills indicating that the ice sheet was 400 m thicker than present before 4 Myr (ref. 24).

For consistency, we recalculate the ages previously obtained from the Grove Mountains^28^,^29^ using a ^10^Be half-life of 1.51 Myr (Supplementary Fig. 10; Supplementary Table 3). Compiled ages indicate that the EAIS in this area was thicker by at least 150–220 m than present, at least once before 3.5±0.7 Myr, and then the ice sheet thinned by ∼50–100 m at ∼1.9±0.9 Myr. Complex exposure histories are found for samples with ^10^Be ages of less than 1.6±0.2 Myr; simple exposure histories predominate for samples with larger exposure ages (Fig. 2b). These results are consistent with the distribution of non-glacial and glacial erosion features in the Grove Mountains. High-altitude locations are characterized by non-glacial weathering features, with glacial modification limited to ∼100 m above the present ice surface^27^,^30^.

### Area 2

**Figure 3:**
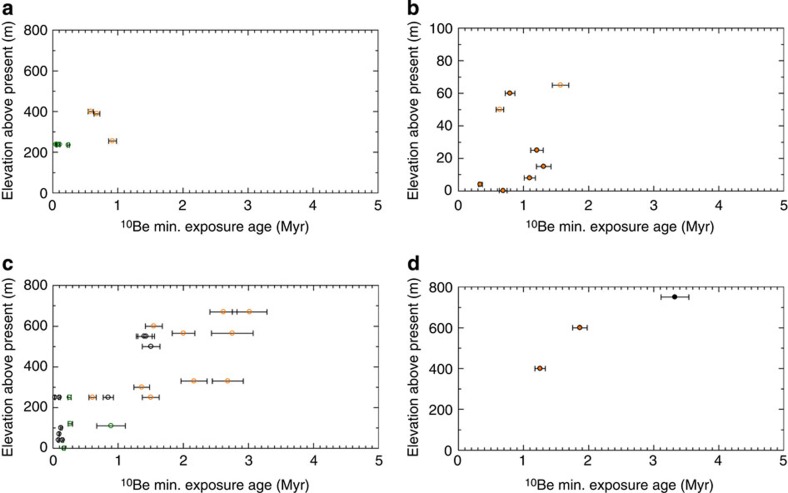
Area 2 ice sheet history. Compiled exposure ages from (**a**) Ricker Hills^31^, (**b**) Allan Hills^25^,^32^, (**c**) Dry Valleys^33^,^34^,^35^ and (**d**) Shackleton Range^36^. In each panel, 1*σ* uncertainty is shown; orange and green symbols indicate simple and complex exposure histories, respectively; black symbols indicate samples lacking ^26^Al measurement; and filled and open symbols refer to bedrock and erratic samples, respectively.

Strasky *et al*.^31^ reported ^10^Be and ^21^Ne exposure ages from the Ricker Hills, VL. The four youngest samples exhibit complex histories, while the older three show simple histories. These data indicate that ice thickness was at least 400 m higher than present at ∼0.6±0.1 Myr (Fig. 3a). This is consistent with glacial erosional and depositional features up to the trimline and with wind erosion at higher altitudes^31^.

Glacial deposits, such as the Sirius Formation, are observed in the nunataks of the Allan Hills^32^. Tschudi *et al*.^32^ reported exposure ages from Sirius Formation boulders and the underlying bedrock. Nishiizumi *et al*.^25^ measured cosmogenic ^10^Be and ^26^Al in rock samples from Allan Hills, which we rescale for differing atmospheric thickness^40^. All samples exhibit a simple exposure history (Supplementary Fig. 11; Supplementary Table 3). Ice thickness in the Allan Hills was at least 70 m higher than the present at ∼1.55±0.15 Myr (Fig. 3b).

Several studies^33^,^34^,^35^ have reported exposure ages from glacial deposits exhibiting differing degrees of weathering (that is, Taylor II–IVb) in the McMurdo Sound-Dry Valleys area of VL, which we compile and rescale^40^ here. Both simple and complex exposure histories are indicated (Supplementary Fig. 12; Supplementary Table 3). We interpret that the ice sheet in this area was thicker by at least 700 m than present, at least once before ∼2.8±0.4 Myr (Fig. 3c). Thickness appears to decrease slightly within the Late Pliocene. From ∼2.2±1.0 Myr, samples exhibit a baseline thickness of ∼300 m. The most recent samples indicate a thickness similar to that of the present day. Complex exposure histories are found for samples with ^10^Be ages of less than 0.9±0.2 Myr, with simple exposure histories predominating for samples with larger exposure ages.

Fogwill *et al*.^36^ reported three ^10^Be and two ^26^Al exposure ages from the horizontal bedrock of a plateau in the Shackleton Range, CL. Both samples with reported ^26^Al concentrations exhibit ^10^Be ages of ∼1 and 2 Myr with a simple exposure history (Supplementary Fig. 13; Supplementary Table 3). These were originally considered to indicate that the ice sheet has been in a relatively stable condition for at least the past 1 Myr. Considering the additional sample that lacks a corresponding ^26^Al measurement and has a much older ^10^Be exposure age (∼3 Myr), we interpret these data to indicate that the EAIS at this location was thicker by at least 750 m than present, at least once before 3 Myr, before thinning by ∼350 m by 1 Myr (Fig. 3d). This is consistent with a meteorite exposure age that indicates that thickness did not exceed 350 m during the past 415 ka and with the inferred late Miocene or Pliocene age of striated till and bedrock at a higher altitude^41^.

### Area 3

**Figure 4:**
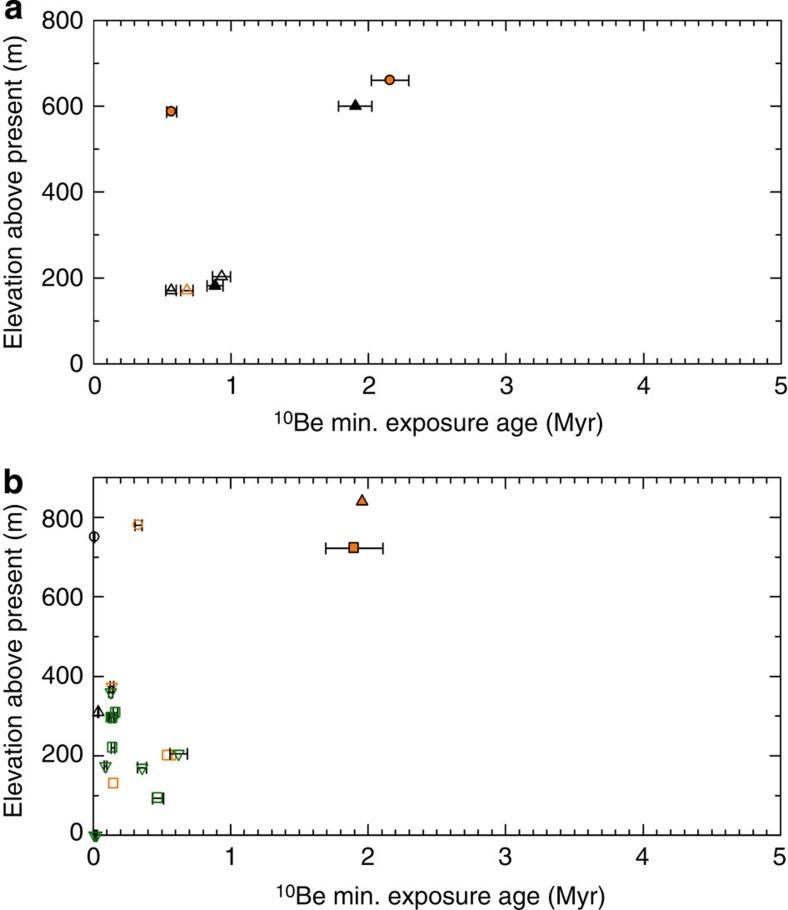
Area 3 ice sheet history. Compiled exposure ages from (**a**) the Fisher Massif (triangles) and Amery Oasis (circles) in the Prince Charles Mountains^37^, and (**b**) the Black Ridge (circles), Mt. Keinath (triangles), Mt. Abbott (squares) and Mt. Browning (inverted triangles) in the Deep Freeze Range^38^,^39^. Systematic uncertainties of ±1*σ* are also indicated. Orange and green symbols indicate simple and complex exposure history, respectively, and black symbols indicate samples with only ^10^Be measurement. Filled and open symbols refer to bedrock and erratic samples, respectively.

Fink *et al*.^37^ presented a deglacial chronology based on seven cosmogenic ^10^Be and ^26^Al exposure ages from three areas of the northern Prince Charles Mountains, MRL. All data exhibit a simple exposure history (Supplementary Fig. 14; Supplementary Table 3). The elevation of the EAIS in both the Fisher Massif and the Amery Oasis was at least 650 m higher than present at ∼2.15±0.15 Myr (Fig. 4a). The elevation of the ice sheet in the Fisher Massif remained relatively unchanged until ∼0.5 Myr, but that of the Amery Oasis thinned to ∼400 m between ∼2 and ∼1 Myr.

Oberholzer *et al*.^38^ and Nicola *et al*.^39^ reported exposure ages from glacially rounded bedrock and erratics obtained from nunataks of the Deep Freeze Range (Supplementary Fig. 8). Complex exposure histories are not observed at any site until after ∼0.6±0.1 Myr (Supplementary Fig. 15; Supplementary Table 3). At ∼1.9±0.2 Myr, ice sheet thickness was at least 750–850 m higher than the present (Fig. 4b). The ice sheet at Mt. Keinath and Mt. Abbott thinned from ∼1.9±0.2 Myr. Exposure at the Black Ridge and Mt. Browning occurred in the Mid Pleistocene. The young exposure age (∼11 ka) of an erratic at a 950-m altitude has been interpreted to indicate potential shielding or post depositional overturning and should be interpreted with caution^38^. These results are consistent with weathered nunatak summits that are free of glacial deposits^38^,^39^.

### Exposure age reconstruction of EAIS fluctuations

As illustrated in the above compilation, there is a wide range in reconstructed thickness across each of the sites of Areas 1–3, which indicates between 150 and 800 m increase in thickness relative to present, at least once before ∼4 Myr. The variability in thickness is similar to the differing altitudes of each sampling location. For example, the maximum altitude of the Grove Mountains is ∼200 m above the present height of the EAIS, making this region unable to record any additional increase in thickness. Individual ages appear to range over 1–2 Myr period over the Pliocene, with an average ∼1-Myr age uncertainty. Except for the >4-Myr age, all error bars (±1*σ*) are overlapping such that we cannot discern any significant age differences. Nonetheless, we acknowledge that these are local sites distributed over a wide spatial area, and there is the possibility that some of these ages reflect local dynamics. Considered individually, however, each of the areas exhibits an overall decrease in thickness towards the present.

To explore the overall Plio-Pleistocene EAIS fluctuations, we consider conceptual ice sheet profiles for specific time slices. This exercise indicates that thinning likely began in inland Area 1 between 3 and 2.5 Myr, followed by a similar degree of thinning in Area 2 between 2.5 and 2 Myr. The onset of thinning in Area 3, proximal to the ice sheet edge, appears to have been delayed until ∼2 Myr. Further thinning to near present levels likely occurred in all areas between 1 and 0.6 Myr.

### Modelled reconstruction of EAIS fluctuations

**Figure 5:**
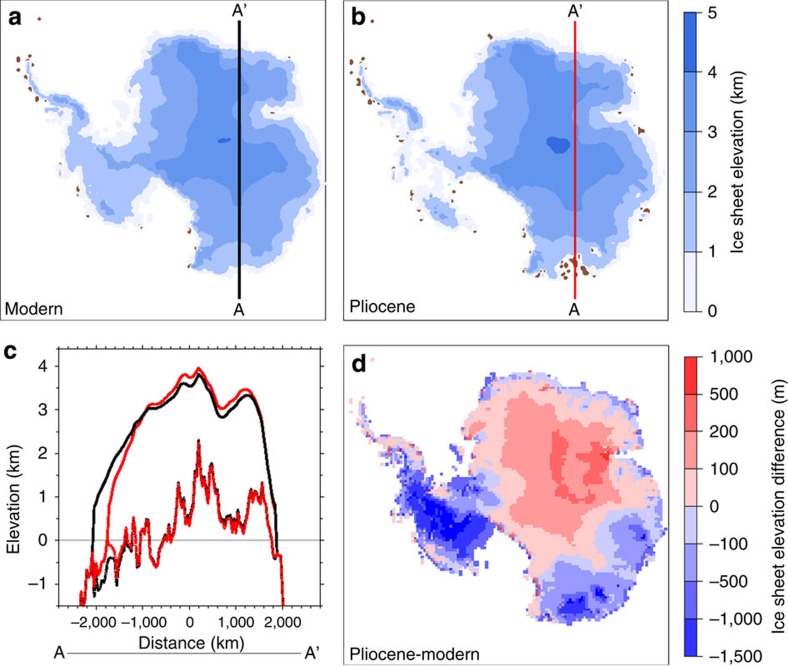
IcIES-modelled antarctic ice sheet. Modelled ice sheet elevation under (**a**) modern and (**b**) Pliocene boundary conditions. (**c**) Cross-section of ice sheet (solid) and bedrock (dashed) elevations from A to A′ for the modern (black) and Pliocene (red) result. (**d**) Pliocene-modern ice sheet elevation.

We utilize the Ice sheet model for Integrated Earth system Studies (IcIES)^42^,^43^ (Fig. 5) to further examine Pliocene EAIS thickness and to independently assess the potential complications of constructing idealized ice sheet profiles from widely distributed exposure age data. Modern (pre-industrial) and Pliocene ice sheet experiments assume equilibrium response of the Antarctic ice sheet under their respective climatic conditions. The main climatic differences considered here are larger precipitation and higher temperature during the Pliocene, primarily due to increased CO_2_ concentration relative to preindustrial (405 versus 280 p.p.m., respectively)^44^ (see Methods). Modern initial conditions are based on the current ice-sheet configuration, while Pliocene conditions are approximated^45^ based on information indicating that the ice sheet retreated more often in the Pliocene than the late Pleistocene.

For the Pliocene, the model produces an ∼−3-m SLE change in the WAIS volume. The ice sheet interior in East Antarctica is generally thicker, consistent with the individual exposure age sites (for example, Figs 1 and 5), as well as with the conceptual ice sheet profiles. However, the ice sheets covering the Wilkes Subglacial and Aurora Basins thin and retreat inland by several hundred kilometres (Fig. 5c). Therefore, in total the modelled EAIS contribution to the Pliocene sea level is ∼0 m SLE because increased interior thickness largely offsets the coastal retreat and collapse of large portions of the Wilkes Subglacial and Aurora Basins, where bed topography is relatively low in East Antarctica.

## Discussion

**Figure 6:**
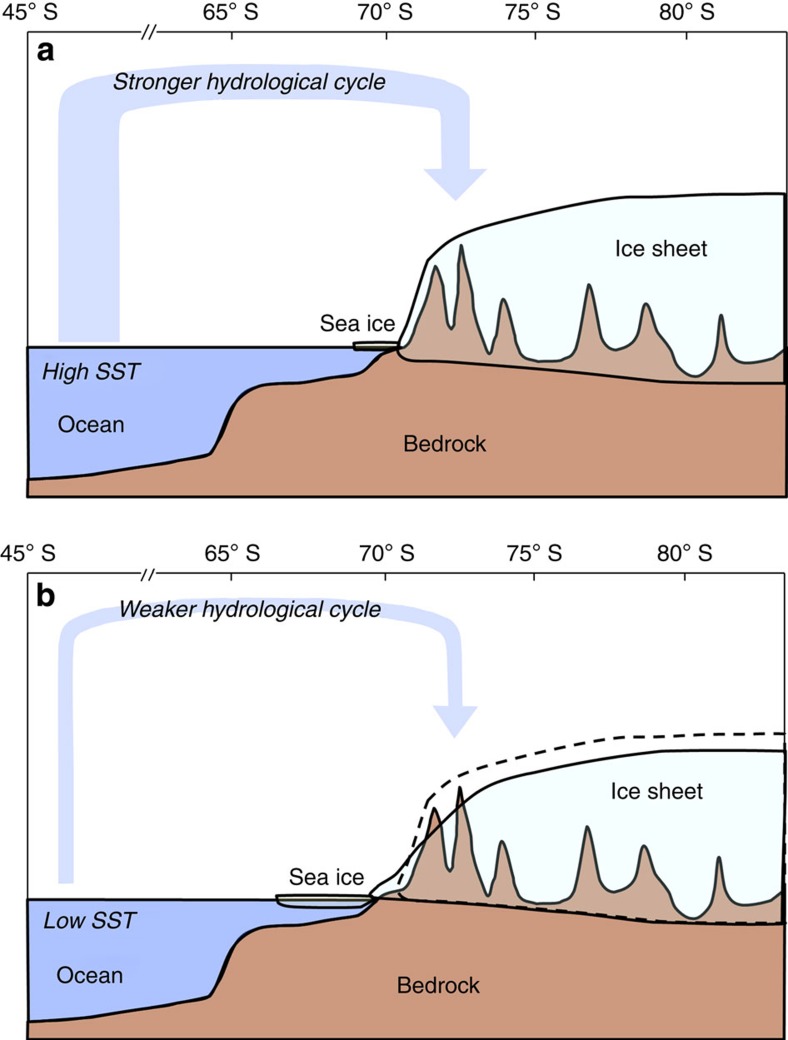
Schematic diagram of ice sheet–climate interactions. (**a**) The Early-Mid Pliocene (>3.0 Myr) and (**b**) the Mid-Late Pleistocene (<1.0 Myr). Dashed line represents the Antarctic ice sheet surface during the early Pliocene.

Thickening of the EAIS by up to 600 m before 3 Myr (Fig. 6) at first seems to contradict inferred relative high Pliocene sea level. On the basis of present ice volumes, complete melting of the Greenland Ice Sheet and the WAIS obtains ∼7 and 6 m SLE, respectively, indicating that an additional ∼7 m SLE would be required to reconcile a potential +20 m Pliocene sea level^2^. Our model produces the expected ∼3-m SLE decrease in volume of the WAIS, but almost no SLE decrease in that of the EAIS, where interior thickening largely offsets the near complete melting of the Wilkes Subglacial and Aurora Basins (which are unsuitable for exposure dating due to the lack of unglaciated area; Fig. 5). This is similar to the coastal retreat and interior thickening currently observed by satellite in Greenland and Antarctica under anthropogenic warming^46^,^47^ and supports the IPCC AR5 interpretation that a value of +20 m is a maximum on Milankovitch timescales (for example, greater than ∼21 k.y., although it does not rule out shorter perturbations). The full range of reconstructed positive Pliocene sea level excursions spans 5–50 m (refs 44, 48, 49, 50). Our results and the ±10-m uncertainty of benthic *δ*^18^O reconstructions indicates that the lower end is most likely.

Other major basins can be eliminated as large contributors to the Pliocene sea level on the basis of our model results, and increased thickness is observed in both our model- and nuclide-based reconstructions. Similar to the WAIS, which is inferred to have retreated during the Pliocene^15^, the Wilkes Subglacial Basin is grounded below the sea level. Geological and geochemical evidence indicate that its behaviour is also dynamic^19^,^20^, and a recent provenance study indicates that offshore Pliocene detrital sediment is sourced from Wilkes Land^19^. These results highlight the importance of bedrock elevation to ice sheet susceptibility to melting and retreat.

A thicker Pliocene EAIS was likely the result of enhanced moisture transport, resulting in increased upstream snowfall, as opposed to higher temperatures^16^, and is consistent with higher atmospheric pCO_2_ (refs 5, 7, 8), and global sea surface temperature (+ ∼2 to 3 °C)^3^,^4^,^5^,^6^. Higher SST and decreased sea ice would have induced more evaporation from the ocean, increasing transport of water vapour to Antarctica that in turn created a thicker EAIS (Fig. 6a). This scenario is in line with the argument put forward by Prentice and Matthews^51^ that the sea-surface temperature of the Southern Ocean controls snowfall at Antarctica (snow gun hypothesis). Modern satellite observations indicate thickening of the interior EAIS in response to increased snowfall^52^.

The interpretation of more effective heat transport and a stronger hydrologic cycle in the Southern Ocean before 3 Myr is supported by results from Atmosphere-Ocean General Circulation Model experiments that indicate the global mean annual SST at 3 Myr was 3 °C higher than today^53^. A Recent model comparison study suggests that the uncertainty between different models is large in the high-latitude Southern Hemisphere^54^. In addition, General Circulation Model results indicate an overall 5% increase in precipitation during the Pliocene^55^, with an increase in the mean annual total precipitation rate (0.2–2 mm per day) in coastal Antarctic regions relative to today^56^.

After 3 Myr, EAIS thickness decreased as global climate cooled and Northern Hemisphere Ice Sheets expanded. We interpret this to be primarily caused by decreased SST that resulted in reduced moisture transport to Antarctica (Fig. 6b). Therefore, the net transport of humidity from the lower latitude ocean to Antarctica may have decreased, reducing snowfall^28^,^30^,^57^.

During the Pliocene, coastal areas of the EAIS retreated and the interior thickened, similar to modern ice sheets under anthropogenic warming. Some areas of the EAIS were up to 600 m thicker than present at least once before 3 Myr. It is plausible that increased interior thickness largely offsets the decrease from melting in Wilkes Land, primarily from the Wilkes Subglacial and Aurora Basins, resulting in little change in EAIS volume. Results presented here are consistent with the IPCC AR5 maximum estimate and suggest a ∼10-m Pliocene sea level increase on Milankovitch Cycle timescales.

## Methods

### Chemical procedures and measurements of cosmogenic nuclides

Chemical procedures to prepare samples for ^10^Be and ^26^Al measurements were on the basis of those of Kohl and Nishiizumi^58^, modified for processing gneiss samples. Chemical processing was performed in three steps: crushing of rock samples and purification of quartz, dissolution of quartz, and isolation and purification of beryllium and aluminium. Separation of ^10^Be and ^26^Al from rock samples was performed at the University of Tokyo.

Prepared samples were then measured using accelerator mass spectrometry (AMS) at the University of Tokyo^59^. The standards used were KNB5–1 for ^10^Be (^10^Be/^9^Be ratio 2.997 × 10^−11^; (ref. 60)) and KNA4-2 for ^26^Al (^26^Al/^27^Al ratio 3.029 × 10^−11^; (ref. 61)). The background levels typically ranged from 1.32 × 10^−14^ to 4.66 × 10^−14^ for the ^10^Be/^9^Be measurements and from 5.0 × 10^−15^ to 6.91 × 10^−15^ for ^26^Al/^27^Al. Half-lives used were 1.51 Myr for ^10^Be (ref. 60) and 0.70 Myr for ^26^Al (ref. 61). The more recent value of 1.34 Myr ^10^Be half-life was not used because of the lack of raw data needed to recalculate the ages from older studies. Our conclusions are not significantly altered with a 1.34-Myr ^10^Be half-life. Nuclide production rates at each sampling site were calculated using the conventional production rates (5.1±0.3 atoms g^−1^ yr^−1^ for ^10^Be and 31.1±1.9 atoms g^−1^ yr^−1^ for ^26^Al).

### Antarctic atmospheric pressure

Because nuclide production rate scales with atmospheric thickness^40^, we accounted for the effect of potential differences in Antarctic atmospheric pressure between the Pliocene and modern. (This effect is most pronounced when nuclide concentration is high.) We applied a scaling factor to nuclide concentrations reported here and, when appropriate, also to the compiled data. The scaling is on the basis of the assumption that the warm Pliocene atmospheric pressure was 2–3 hPa higher than the present (see Supplementary Table 4 and Supplementary Note 4). This results in a typically small (0.2%) change in the production rate that causes little change in the calculated exposure age. These final derived site production rates were employed to deduce exposure ages for each location.

### IcIES ice sheet model

The numerical experiments were performed using the Ice sheet model for IcIES, as described in refs 42, 43), with several modifications explained below. Boundary conditions such as bedrock topography, surface temperature and accumulation were replaced with those provided by the Sea-level Response to Ice Sheet Evolution project^62^. The atmospheric lapse rate is assumed to be uniform at 8 K km^−1^. The enhancement factor and the geothermal heat flux were assumed to be uniform and, respectively, retuned to 3 and 54.6 mW m^−2^ to simulate the volume of the present-day Antarctic ice sheet. In addition, the basal sliding parameterization was revised to allow submelt sliding following ref. 63. The same Weertman-type parameterization^64^ is adopted for basal sliding with the coefficient (14.4 × 10^−10^ m^8^ yr^−1^ N^−3^), which is eight times larger than that used in ref. 42. IcIES was run on a 40 × 40-km grid with 26 vertical layers. The time step was 0.5 year to stabilize the solution to larger basal velocities. Ice shelf processes are not included, and the ice sheet portion was only calculated with the shallow ice approximation, which is most important when the EAIS change is focused for the Pliocene situation. The lateral boundary is defined by floating conditions; ice that becomes afloat is cut off immediately^65^.

Two sets of experiments using IcIES were conducted, one for the ‘modern' (Pre-Industrial) and the other for the ‘Pliocene' situation using different climatic conditions and initial conditions, assuming the Antarctica ice sheet responds to equilibria under different climatic conditions. The climatic anomaly difference was obtained and simplified from the MIROC Pliocene experiment^66^, which was used to conduct the experimental set-up following the Pliocene model intercomparison project (PlioMIP^44^,^67^). The main climatic difference assumed in the Pliocene experiments of PlioMIP was in the atmospheric level of CO_2_, 405 versus 280 p.p.m. (Pliocene and pre-industrial level, respectively) as well as differing biome distributions, land sea mask and ice sheet configuration/topography on the basis of the PRISM3 data set^44^,^68^,^69^. Since the assumption in the PlioMIP experiment includes a different ice sheet shape (smaller in the Pliocene), the modelled climate of Atmosphere-Ocean General Circulation Model (PlioMIP) is strongly influenced by the assumed size and shape of ice sheet (retreated in the West Antarctica, Willkes Basin and Aurora Basin) and induces a large inhomogeneity of climatic conditions, that is, very warm and moist climate over the ice-retreated area through topographic and albedo feedback. To avoid this inhomogeneous climate change caused by these assumptions, we assume a homogeneous anomaly of temperature and precipitation rate by averaging the difference of MIROC between Pliocene and pre-industrial experiments. The temperature change due to topography change was subtracted. As a result, the climatic inputs for the ice sheet model are a temperature increase of 3 °C and a precipitation ratio increased by 50% in the Pliocene experiment compared with the pre-industrial one. The initial conditions for the modern and Pliocene ice sheets are those observed for present day (on the basis of BEDMAP2) and PRISM ice sheet from an approximation of the Pliocene ice sheet^45^,^68^. As a result, the Pliocene ice sheet extent is much larger than as initially specified, indicating that the model is not so sensitive to the specific initial conditions. Rather, the separation of Eastern and West Antarctica is more important. Each experiment was performed for 200,000 years in order to obtain steady-state solutions (Supplementary Fig. 16).

## Author contributions

Y.Y., M.Y. and A.A.-O. designed the research; K.M. collected the rock samples; M.Y. and H.M. carried out AMS measurements; M.Y. compiled and recalculated exposure ages. All authors contributed to the interpretation of the data. A.A.-O. and F.S. performed model simulations. S.O., M.Y., Y.Y. and A.A.-O. wrote the paper together with input from all other authors.

## Additional information

**How to cite this article:** Yamane, M. *et al*. Exposure age and ice-sheet model constraints on Pliocene East Antarctic ice sheet dynamics. *Nat. Commun*. 6:7016 doi: 10.1038/ncomms8016 (2015).

## Supplementary Material

Supplementary Information Supplementary Figures 1-16, Supplementary Tables 1-4, Supplementary Notes 1-4 and Supplementary References
