# Supplementary material for: Exposure age and ice-sheet model constraints on Pliocene East Antarctic ice sheet dynamics
Source: Nat Commun. 2015 Apr 24;6:7016. doi: 10.1038/ncomms8016 (PMC4421805; doi:10.1038/ncomms8016)
Supplement: Supplementary Information — Supplementary Figures 1-16, Supplementary Tables 1-4, Supplementary Notes 1-4 and Supplementary References [file ncomms8016-s1.pdf]

Supplementary Figures

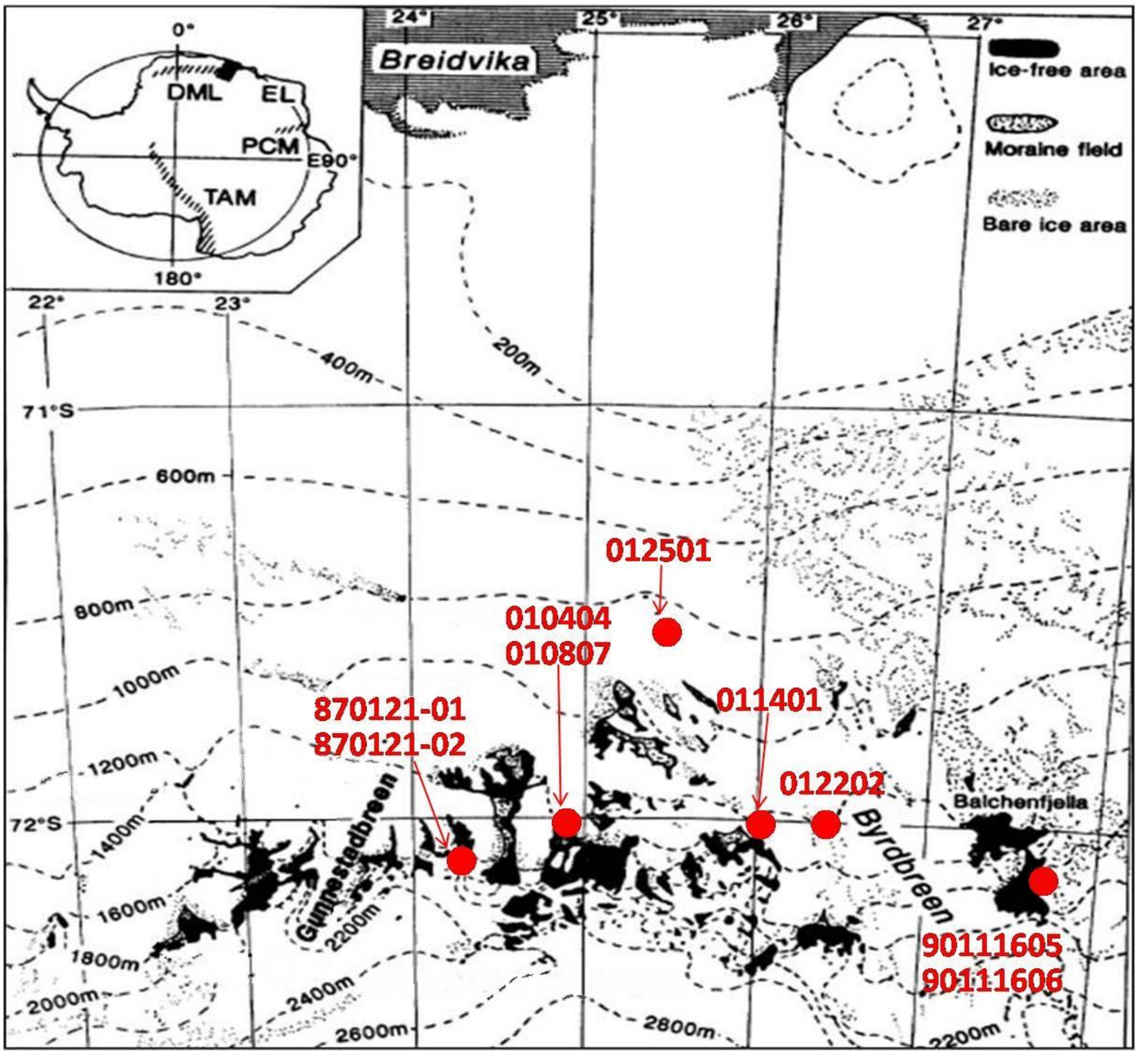

Supplementary Figure 1. Modified Location map[1] of the Sør Rondane Mountains. Red dots show sampling sites of this study.

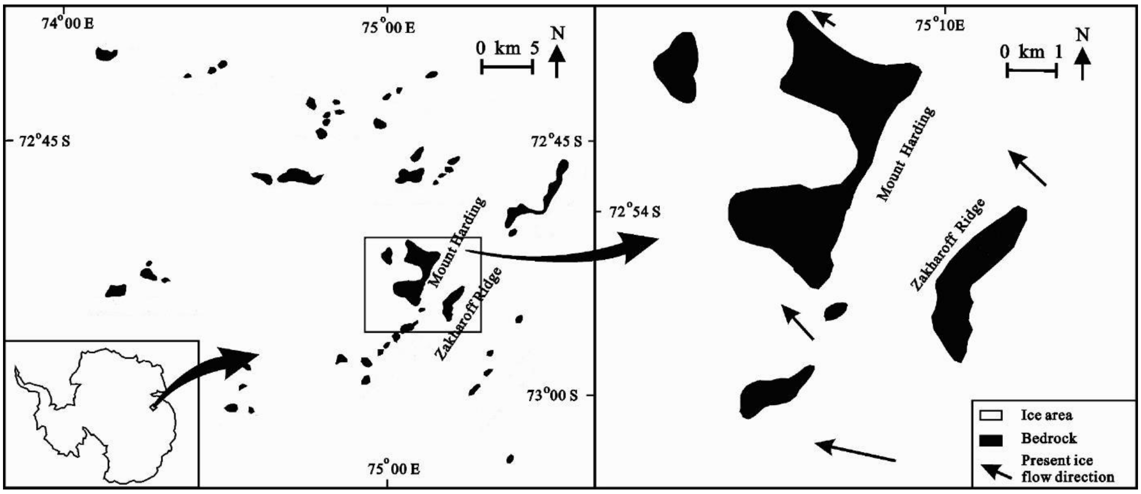

Supplementary Figure 2. Modified Location map of Groove Mountain[2].

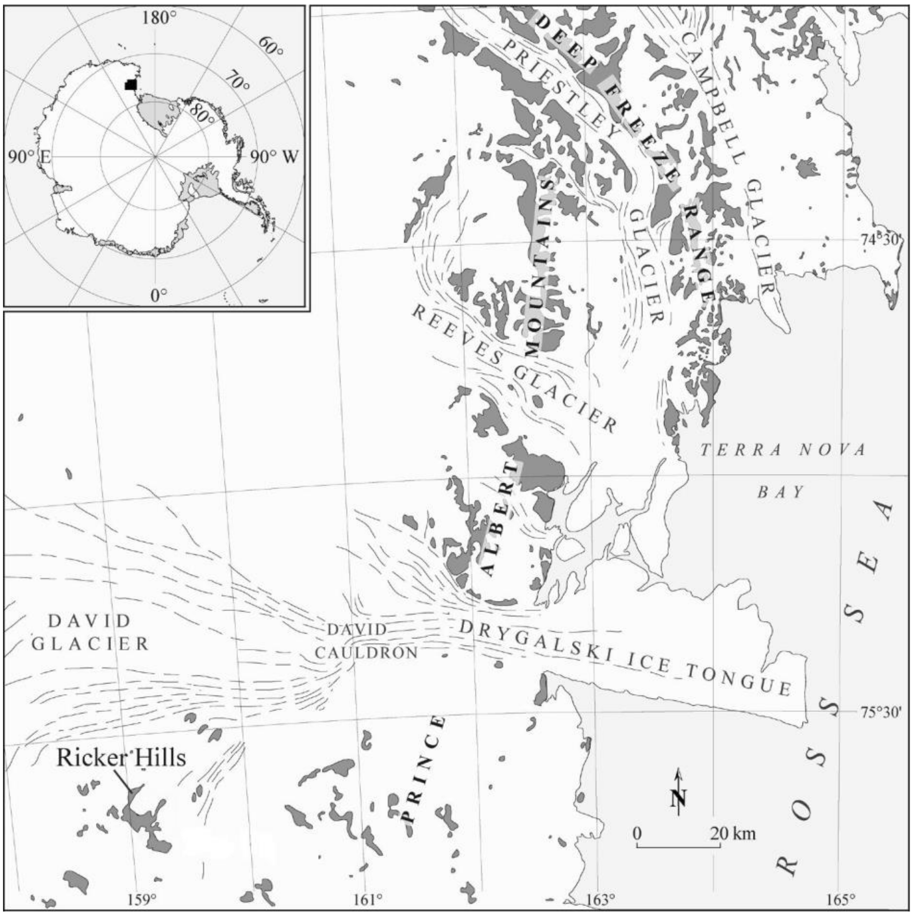

Supplementary Figure 3. Modified Location map of the Ricker Hills[3].

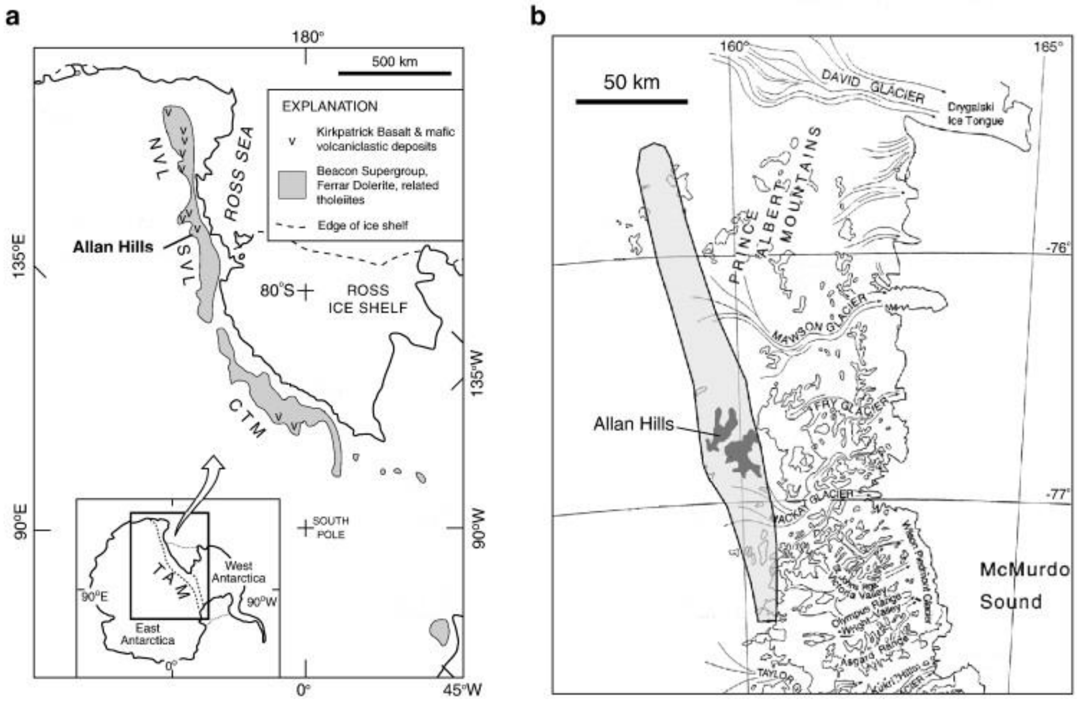

Supplementary Figure 4. Modified Location map of the Allan Hills[4].

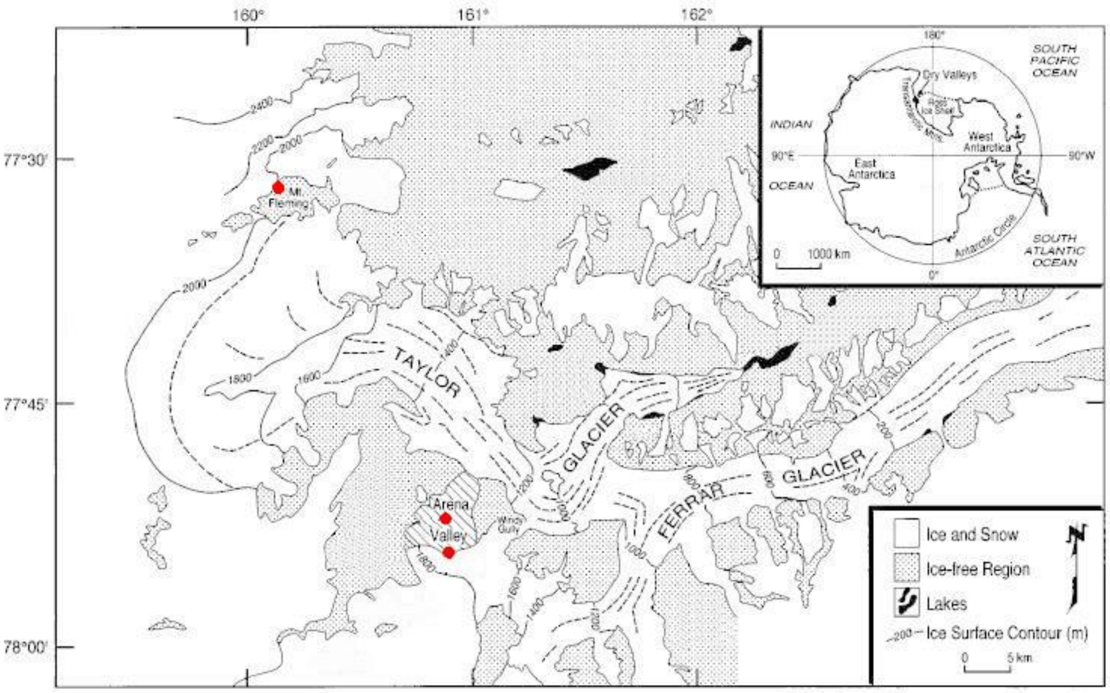

Supplementary Figure 5. Modified Location map of the Dry Valleys[5]. Red dots show sampling sites.

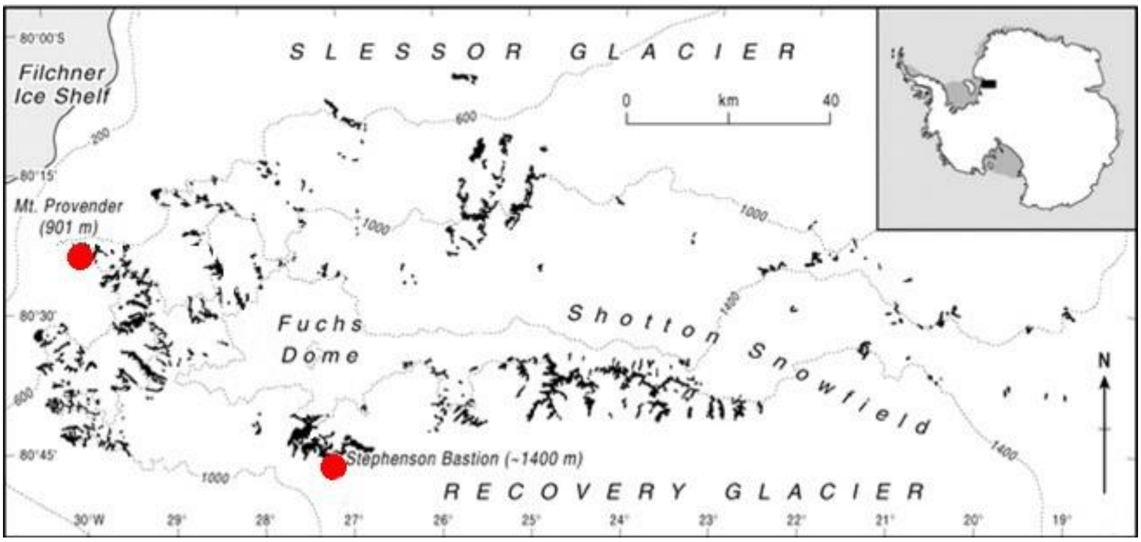

Supplementary Figure 6. Modified Location map of Shackleton Range[6]. Red dots show sampling sites

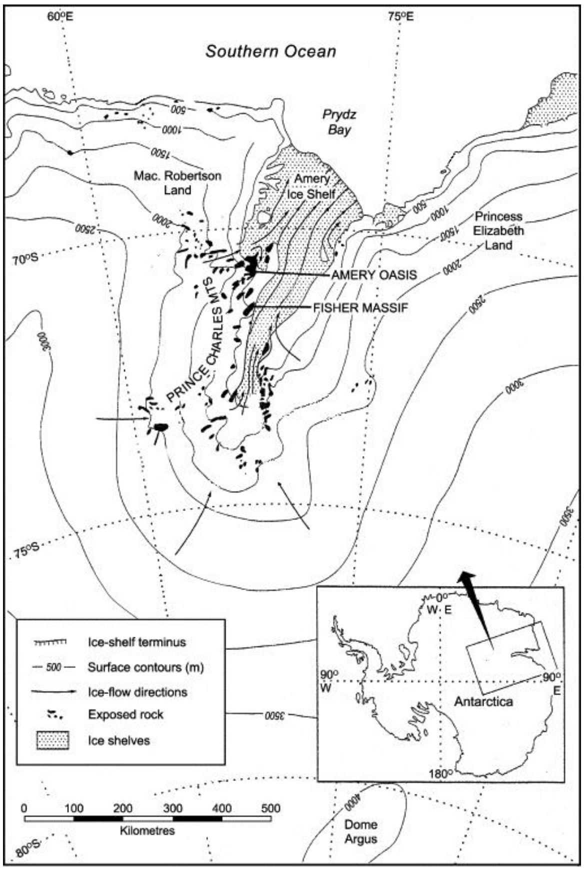

Supplementary Figure 7. Modified Location map of Prince Charles Mountains[7].

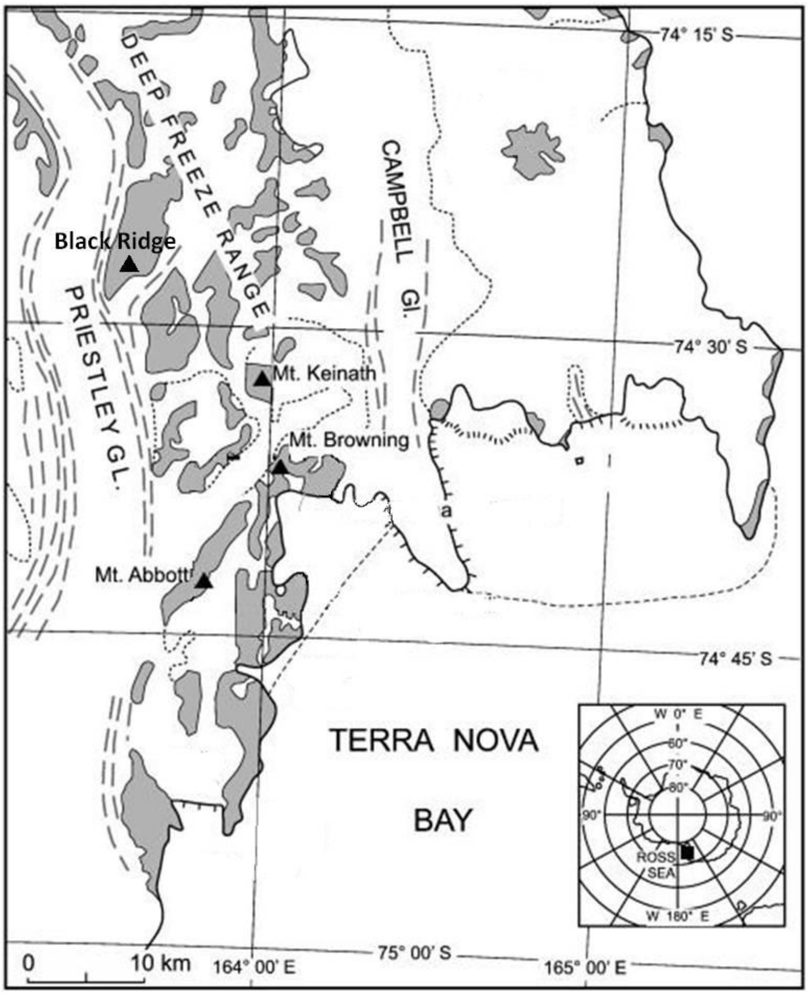

Supplementary Figure 8. Modified Location map of Deep Freeze Range[8].

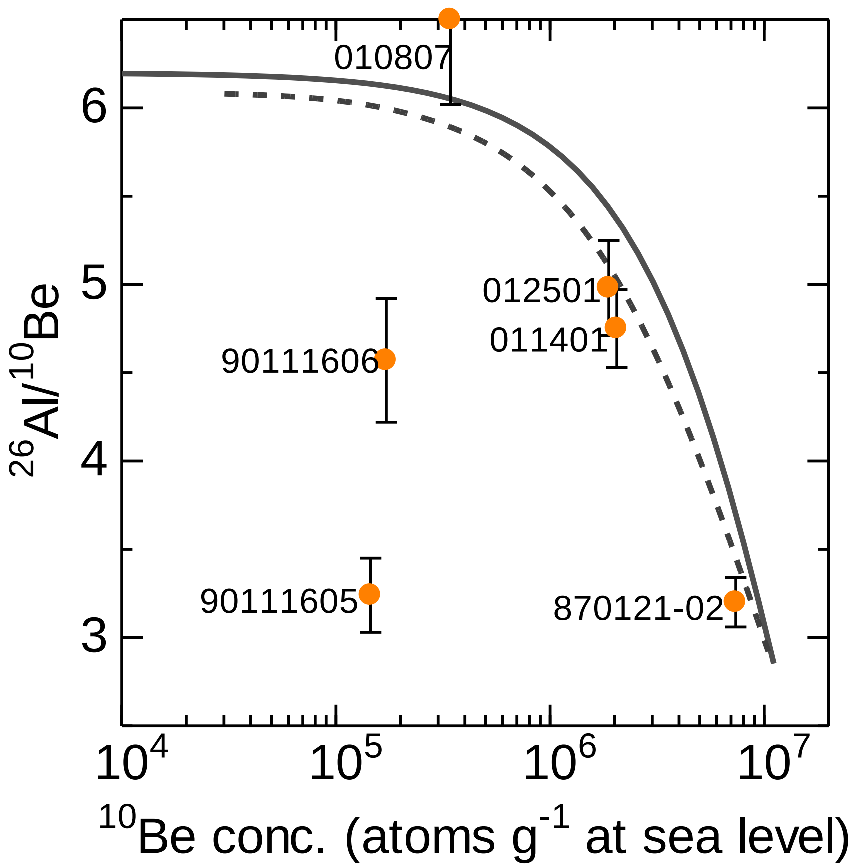

Supplementary Figure 9. Sør Rondane ^{26}Al/^{10}Be ratio plotted against ^{10}Be concentrations. Uncertainties (1σ) are also indicated. The solid line indicates the ^{26}Al/^{10}Be ratio assuming a simple exposure history, while the dashed line assumes steady-state erosion.

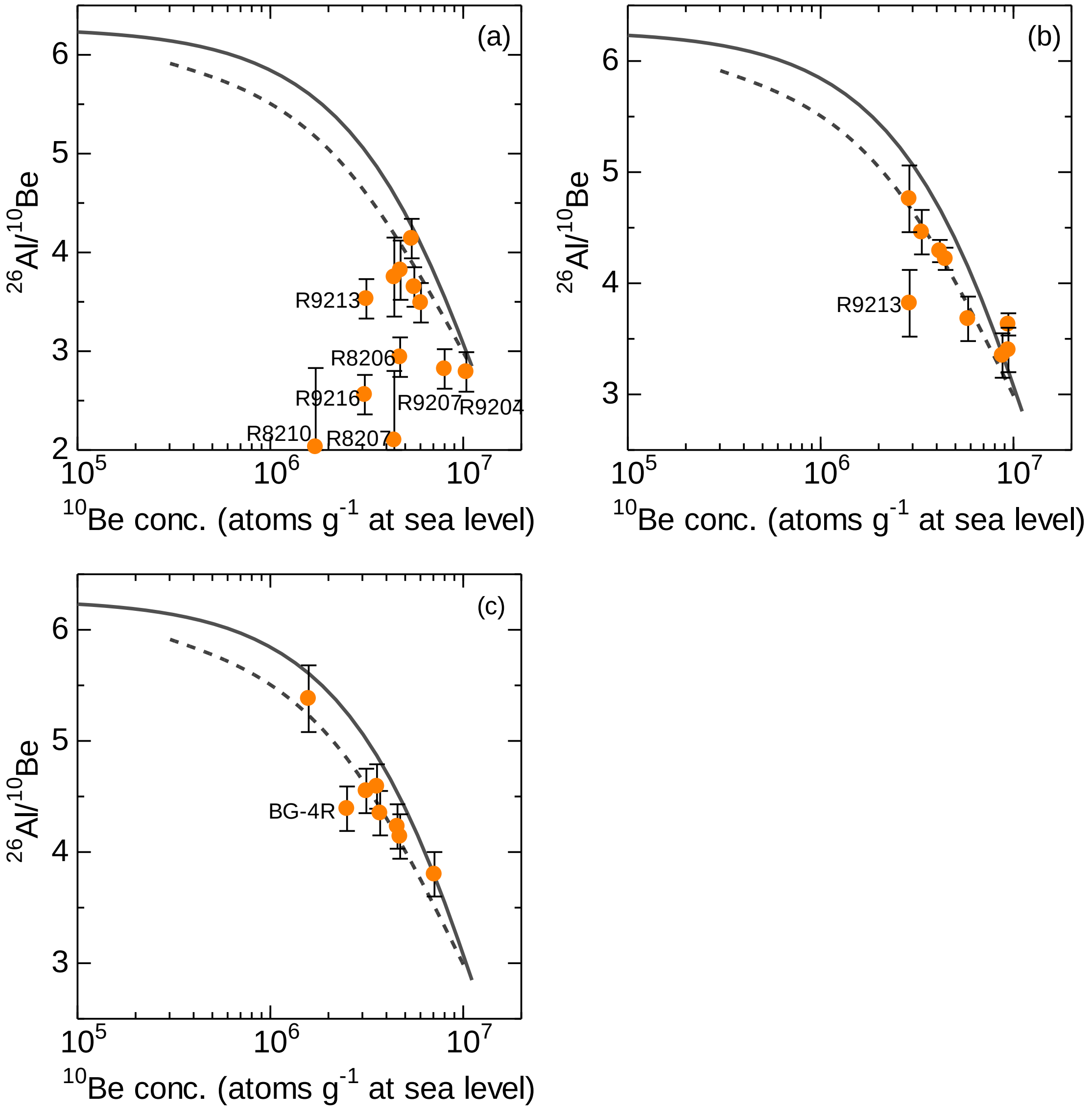

Supplementary Figure 10. Grove Mountains $^{26}Al/^{10}Be$ ratio plotted against ^{10}Be concentrations. Uncertainties (1σ) are also indicated. The solid line indicates the $^{26}Al/^{10}Be$ ratio assuming a simple exposure history, while the dashed line assumes steady-state erosion. (a) cosmogenic data from ref. 2, (b) data from ref. 9 and 10, and (c) data from ref. 11.

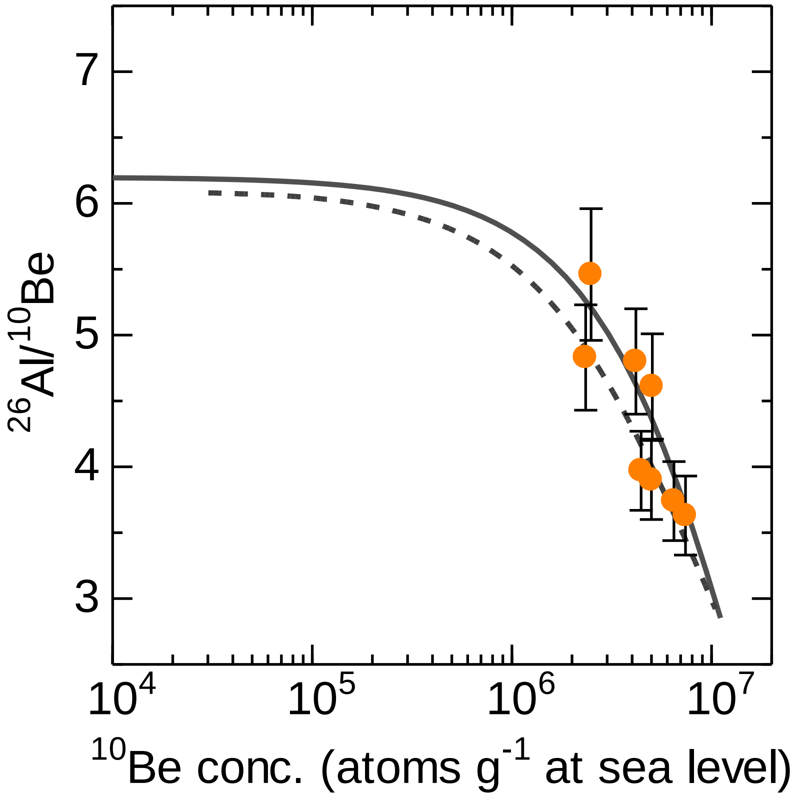

Supplementary Figure 11. Allan Hills[12,13] $^{26}Al/^{10}Be$ ratio plotted against ^{10}Be concentrations. Uncertainties (1σ) are also indicated. The solid line indicates the $^{26}Al/^{10}Be$ ratio assuming a simple exposure history, while the dashed line assumes steady-state erosion.

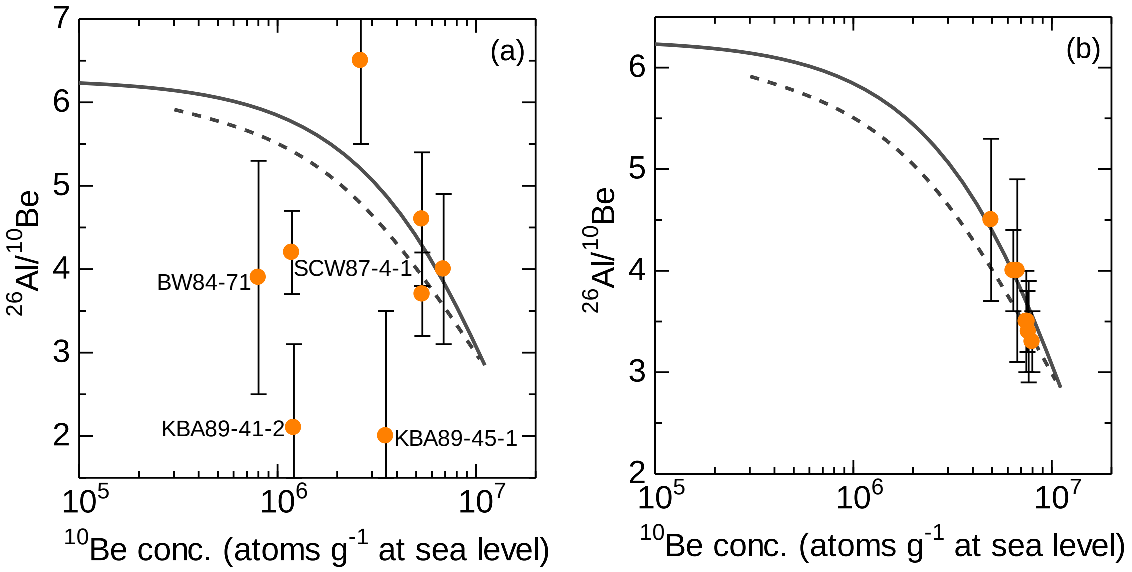

Supplementary Figure 12. Dry Valleys ^{26}Al/^{10}Be ratio plotted against ^{10}Be concentrations. Uncertainties (1σ) are also indicated. The solid line indicates the ^{26}Al/^{10}Be ratio assuming a simple exposure history, while the dashed line assumes steady-state erosion. (a) cosmogenic data from ref. 14, and (b) data from ref. 5.

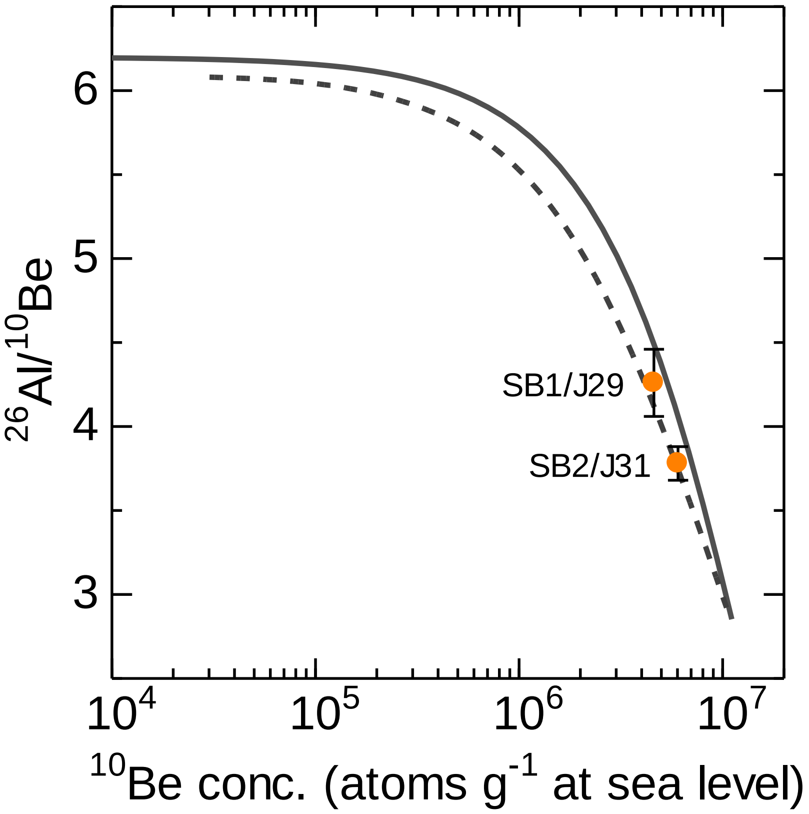

Supplementary Figure 13. Shackleton Range[6] ^{26}Al/^{10}Be ratio plotted against ^{10}Be concentrations. Uncertainties (1σ) are also indicated. The solid line indicates the ^{26}Al/^{10}Be ratio assuming a simple exposure history, while the dashed line assumes steady-state erosion.

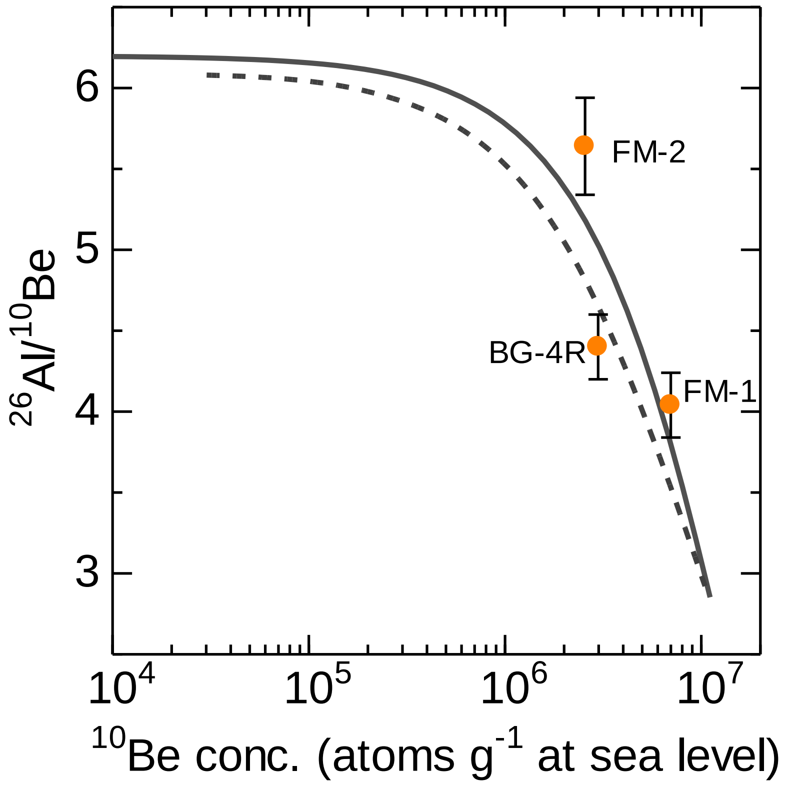

Supplementary Figure 14. Prince Charles Mountains[7] $^{26}Al/^{10}Be$ ratio plotted against ^{10}Be concentrations. Uncertainties (1σ) are also indicated. The solid line indicates the $^{26}Al/^{10}Be$ ratio assuming a simple exposure history, while the dashed line assumes steady-state erosion.

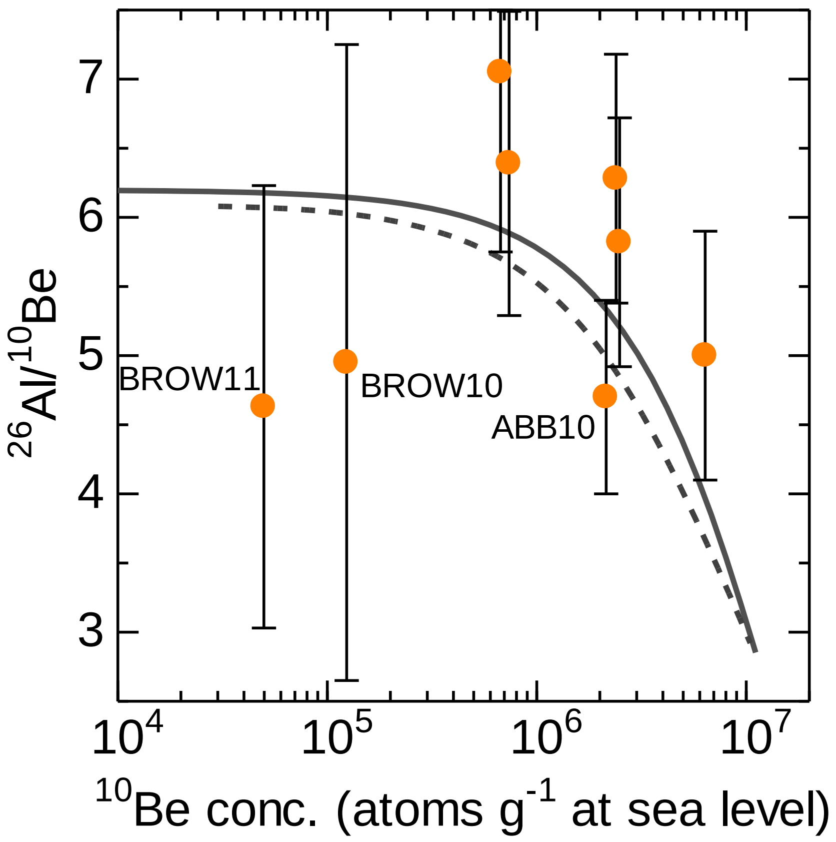

Supplementary Figure 15. Deep Freeze Range[8] $^{26}Al/^{10}Be$ ratio plotted against ^{10}Be concentrations. Uncertainties (1σ) are also indicated. The solid line indicates the $^{26}Al/^{10}Be$ ratio assuming a simple exposure history, while the dashed line assumes steady-state erosion.

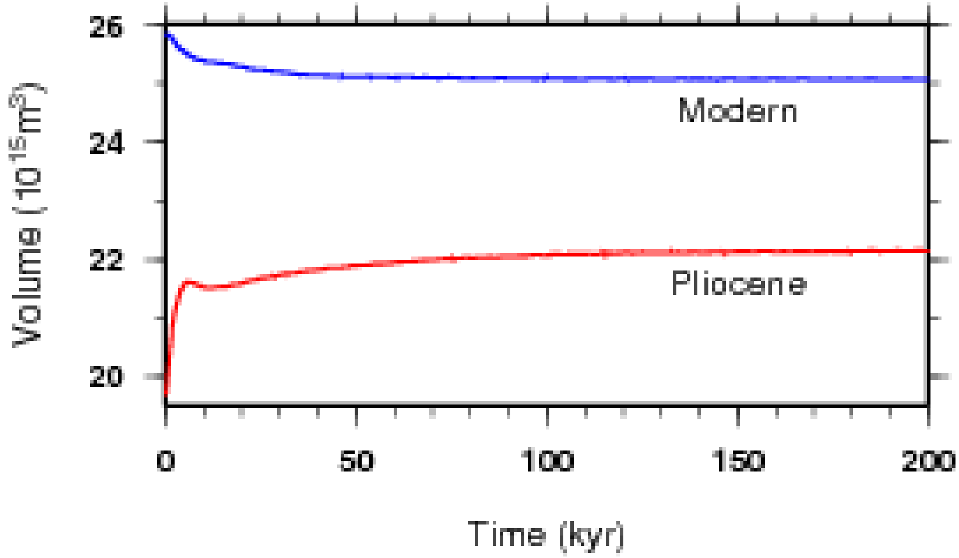

Supplementary Figure 16. Modeled evolution of total ice-sheet obtained with Modern (blue) and Pliocene (red) steady-state experiments.

Supplementary Tables

Supplementary Table 1. Sample locations and cosmogenic nuclide production rates

Sample Name	Sample Type	Latitude	Longitude	Altitude	Above ice	^{10}Be prod. rate			^{26}Al prod. rate		
		(°S)	(°E)	(masl)	(m)	(atoms g^{-1} yr^{-1})			(atoms g^{-1} yr^{-1})		
90111605	Bedrock	72.2	27.8	1600	50	25.75	±	1.51	150.86	±	9.2
90111606	Bedrock	72.2	27.8	1610	60	25.95	±	1.53	152.03	±	9.3
012202	Erratic boulder	72.0	26.4	1440	180	22.71	±	1.34	133.04	±	8.1
012501	Bedrock	71.6	25.4	971	30	15.38	±	0.90	90.07	±	5.5
011401	Bedrock	72.0	26.0	1676	400	27.07	±	1.59	159.93	±	9.8
010404	Bedrock	72.0	24.9	1620	300	25.71	±	1.51	153.21	±	9.4
010807	Bedrock	72.0	24.9	1304	20	20.01	±	1.18	119.21	±	7.3
870121-01	Bedrock	72.1	24.3	2440	200	45.79	±	2.69	275.30	±	16.8
870121-02	Bedrock	72.1	24.3	2525	300	48.83	±	2.87	291.08	±	17.8

* We used a sample density of 2.7 g cm^{-3}, an attenuation length of 160 g cm^{-2}.

Supplementary Table 2. Cosmogenic nuclide concentrations, exposure ages and $^{26}Al/^{10}Be$ ratio

Sample Name	Quartz (g)	^{27}Al conc. (ppm)	^{10}Be conc. ($\times 10^4$ atoms g^{-1})			^{26}Al conc. ($\times 10^4$ atoms g^{-1})			^{10}Be age (ka)			^{26}Al age (ka)			$^{26}Al/^{10}Be$			
90111605	27.8	40.74	83	±	2	248	±	15	33	±	2	17	±	1				
	15.1	N.D.	70	±	2	N.D.			27	±	2	N.D.						
		(average)	*77*	±	*1*				*30*	±	*2*				3.24	±	0.2	complex
90111606	18.6	8.37	91	±	4	417	±	26	35	±	3	28	±	2	4.57	±	0.4	complex
012202	13.5	N.D.	1408	±	30	N.D.			745	±	57	N.D.			N.D.			
012501	14.9	6.46	592	±	14	2946	±	145	428	±	30	395	±	38	4.98	±	0.3	simple
011401	13.8	2.60	1144	±	27	5435	±	216	476	±	34	414	±	37	4.75	±	0.2	simple
010404	14.8	N.D.	1317	±	28	N.D.			594	±	43	N.D.			N.D.			
010807	14.7	2.70	143	±	6	927	±	57	73	±	5	81	±	7	6.50	±	0.5	simple
870121-01	10.0	N.D.	5487	±	120	N.D.			1851	±	116	N.D.			N.D.			
870121-02	22.8	12.08	7483	±	104	23916	±	994	2980	±	180	1696	±	325	3.20	±	0.1	simple

* 90111605 was obtained from two measurements. The results were averaged taken into account the uncertainties associated with AMS measurements.

Supplementary Table 3. Sample locations, ^{10}Be site production rate, ^{10}Be concentrations, ^{10}Be exposure ages and $^{26}Al/^{10}Be$ ratio of compiled data

Sample Name	Type	Altitude (masl)	Above ice (m)	^{10}Be site prod. rate (atoms g^{-1})	^{10}Be conc. ($\times 10^4$ atoms g^{-1})	^{10}Be age (ka)	$^{26}Al/^{10}Be$		Ref.
AREA 1									
Sør Rondane Mountains, Droning Maud Land									
860119-02	B	1410	40	22.74 ± 1.34	314 ± 9	143 ± 10	5.13 ± 0.3	complex	(12)
860128-01B	B	1410	3	22.74 ± 1.34	84 ± 3	37 ± 3	4.09 ± 0.3	complex	(12)
A860116-03-3	B	1180	180	18.84 ± 1.11	2094 ± 61	1555 ± 149	3.92 ± 0.2	simple	(12)
A860118-02B	B	1470	110	23.85 ± 1.40	1577 ± 60	788 ± 66	4.48 ± 0.2	simple	(12)
A860121-06B	B	2650	350	55.19 ± 3.25	8027 ± 193	2399 ± 278	3.37 ± 0.2	simple	(12)
A860122-01	B	1700	400	28.51 ± 1.68	4276 ± 107	2541 ± 308	3.18 ± 0.1	simple	(12)
A860122-05C	B	1800	600	30.74 ± 1.81	5806 ± 134	>4000	2.91 ± 0.1	simple	(12)
91013103	B	1640	300	26.55 ± 1.56	1740	779 ± 55	4.80	simple	(22)
90122904	B	1680	400	27.38 ± 1.61	2160	980 ± 73	4.54	simple	(22)
870121-01	B	2440	200	46.97 ± 2.76	6930	2464 ± 269	3.63	simple	(22)
Grove Mountains, Princess Elizabeth Land									
R8201	B	2256	156	42.57 ± 2.50	5670 ± 150	2059 ± 221	4.14 ± 0.2	simple	(2)
R8203	B	2243	143	42.19 ± 2.48	4890 ± 130	1654 ± 160	3.75 ± 0.4	simple	(2)
R8205	B	2230	130	41.81 ± 2.46	5060 ± 180	1766 ± 187	3.82 ± 0.3	simple	(2)
R8206	B	2204	104	41.07 ± 2.42	4890 ± 120	1723 ± 167	2.94 ± 0.2	complex	(2)
R8207	B	2178	78	40.34 ± 2.37	4520 ± 120	1573 ± 149	2.10 ± 0.7	complex	(2)
R8210	B	2100	0	38.20 ± 2.25	1950 ± 50	582 ± 43	2.03 ± 0.8	complex	(2)
R9201	B	2300	200	43.86 ± 2.58	6310 ± 150	2353 ± 269	3.49 ± 0.2	simple	(2)
R9210	B	2225	125	41.67 ± 2.45	5590 ± 150	2084 ± 226	3.65 ± 0.2	simple	(2)
R9201	B	2300	200	43.49 ± 2.56	6490 ± 88	2516 ± 279	3.35 ± 0.2	simple	(9)
R9202	B	2292	192	43.26 ± 2.54	4708 ± 110	1508 ± 128	4.05 ± 0.2	simple	(9)
R9203	B	2283	183	42.99 ± 2.53	5555 ± 110	1959 ± 187	3.84 ± 0.1	simple	(9)
R9204	B	2275	175	42.76 ± 2.52	7568 ± 121	3646 ± 555	3.30 ± 0.1	simple	(9)
R9205	B	2267	167	42.53 ± 2.50	5236 ± 77	1814 ± 167	3.90 ± 0.1	simple	(9)
GR41	B	2032	103	36.25 ± 2.13	3698 ± 82	1376 ± 121	4.13 ± 0.2	simple	(10)
GR40	B	2057	128	37.21 ± 2.19	4198 ± 92	1589 ± 147	4.17 ± 0.2	simple	(10)
GR33	B	2066	158	37.29 ± 2.19	3296 ± 64	1134 ± 92	3.98 ± 0.2	simple	(10)
GR82	B	2072	212	36.98 ± 2.18	4244 ± 83	1630 ± 150	3.96 ± 0.2	simple	(10)
GR28	B	2231	46	41.14 ± 2.42	2690 ± 60	777 ± 59	4.90 ± 0.3	simple	(10)
GR27	B	2255	70	42.00 ± 2.47	5926 ± 122	2272 ± 249	3.77 ± 0.2	simple	(10)
GR26	B	2301	116	43.89 ± 2.58	6238 ± 131	2302 ± 255	3.84 ± 0.2	simple	(10)
GR25	B	2322	137	44.14 ± 2.60	7515 ± 212	3313 ± 508	3.45 ± 0.2	simple	(10)
R9204	B	2275	175	43.86 ± 2.58	7444 ± 200	3290 ± 497	3.35 ± 0.2	simple	(11)
R9207	B	2250	150	43.11 ± 2.54	7483 ± 224	3471 ± 563	3.40 ± 0.2	simple	(11)
R9213	B	2200	100	41.66 ± 2.45	3409 ± 96	1026 ± 85	3.82 ± 0.3	simple	(11)
R9216	B	2175	75	40.94 ± 2.41	3296 ± 101	1005 ± 85	4.76 ± 0.3	simple	(11)
AREA 2									
Ricker Hills, Victoria Land									
rh06/01	E	1438	238	22.67 ± 1.33	111 ± 6	50 ± 4	—	(c)	(3)
rh06/03	E	1438	238	22.67 ± 1.33	239 ± 7	108 ± 7	—	(c)	(3)
rhp10	E	1435	235	22.62 ± 1.33	515 ± 15	240 ± 16	—	(c)	(3)
rhs1	E	1438	238	22.67 ± 1.33	154 ± 5	69 ± 5	—	(c)	(3)
rhs2	E	1455	255	22.98 ± 1.35	1723 ± 52	919 ± 61	—	(s)	(3)
rhs3	E	1589	389	25.53 ± 1.50	1492 ± 45	680 ± 45	—	(s)	(3)
rh06/06	E	1600	400	25.75 ± 1.51	1317 ± 40	583 ± 39	—	(s)	(3)

Allan Hills, Victoria Land																	
ALH85-1	B	2085	15	37.80	±	2.22	3721	±	231	1310	±	112	3.63	±	0.3	simple	(12)
ALH85-2	B	1955	4	34.44	±	2.03	1072	±	72	336	±	30	4.83	±	0.4	simple	(12)
ALH85-3	B	2150	60	39.56	±	2.33	2627	±	192	792	±	74	3.90	±	0.3	simple	(12)
ALH85-4	B	1807	8	30.90	±	1.82	2661	±	134	1096	±	85	3.74	±	0.3	simple	(12)
ALH85-9	B	1645	0.2	27.34	±	1.61	1613	±	100	688	±	59	3.97	±	0.3	simple	(12)
227	E	1730	50	29.16	±	1.72	1610	±	110	637	±	57	5.46	±	0.5	simple	(13)
AL9704	E	1745	65	29.50	±	1.74	3300	±	180	1570	±	126	4.61	±	0.4	simple	(13)
AL9711c	B	1705	25	28.62	±	1.68	2650	±	140	1206	±	95	4.80	±	0.4	simple	(13)
McMurdo Sound–Dry Valleys, Victoria Land																	
BW84-87	E	1300	250	20.28	±	1.19	61	±	7	30	±	4		—			(14)
SCW87-4-1	E	1300	250	20.54	±	1.21	490	±	40	253	±	25	4.20	±	0.5	complex	(14)
BW84-134	E	1300	250	20.46	±	1.20	200	±	20	100	±	12		—			(14)
BW84-33	E	1300	250	20.54	±	1.21	1090	±	80	608	±	57	6.50	±	1.0	simple	(14)
BW84-71	E	1050	0	16.60	±	0.98	270	±	20	169	±	16	3.90	±	1.4	complex	(14)
SCW87-5	E	1300	250	19.78	±	1.16	1390	±	100	849	±	79		—			(14)
KBA89-41-2	E	1170	120	18.06	±	1.06	450	±	50	265	±	33	2.10	±	1.0	complex	(14)
KBA89-45-1	E	1160	110	17.76	±	1.04	1300	±	310	892	±	219	2.00	±	1.5	complex	(14)
AA86-14	E	1550	500	25.40	±	1.49	2760	±	190	1505	±	136		—			(14)
AA86-15	E	1600	550	25.53	±	1.50	2640	±	170	1402	±	122		—			(14)
BW84-105	E	1300	250	20.46	±	1.20	2220	±	140	1502	±	130	4.60	±	0.8	simple	(14)
SCW87-3-1	E	1600	550	25.75	±	1.51	2700	±	180	1430	±	127		—			(14)
AA86-16	E	1650	600	26.53	±	1.56	2940	±	180	1548	±	131	3.70	±	0.5	simple	(14)
KBA89-70-1	E	1120	70	17.92	±	1.05	168	±	11	96	±	8		—			(15)
KBA89-96	E	1150	100	18.37	±	1.08	219	±	16	123	±	11		—			(15)
KBA89-99	E	1090	40	17.47	±	1.03	147	±	14	86	±	10		—			(15)
KBA89-97	E	1090	40	17.47	±	1.03	248	±	16	147	±	13		—			(15)
SCW87-1-1	E	1380	330	21.64	±	1.27	2970	±	210	2165	±	199	4.00	±	0.9	simple	(5)
KBA89-108	E	1380	330	21.64	±	1.27	3340	±	220	2684	±	237	3.50	±	0.3	simple	(5)
KBA89-107	E	1350	300	21.13	±	1.24	2140	±	140	1363	±	120	4.50	±	0.8	simple	(5)
BAK90-77	E	1720	670	28.22	±	1.66	4610	±	300	3018	±	265	3.30	±	0.3	simple	(5)
BAK90-73-1	E	1720	670	28.22	±	1.66	4300	±	230	2618	±	208	3.50	±	0.5	simple	(5)
BAK90-32	E	2065	565	36.33	±	2.14	4760	±	300	2004	±	173	4.00	±	0.4	simple	(5)
BAK90-32-1	E	2065	565	36.33	±	2.14	5680	±	570	2754	±	320	3.40	±	0.5	simple	(5)
Shackleton Range, Coats Land																	
SB1/J29	B	1600	400	25.35	±	1.49	2420	±	60	1256	±	80	4.26	±	0.2	simple	(6)
SB2/J31	B	1800	600	29.51	±	1.74	3700	±	40	1867	±	112	3.78	±	0.1	simple	(6)
NL1/J22	B	950	750	14.25	±	0.84	2430	±	60	3326	±	212		—			(6)
AREA 3																	
Prince Charles Mountains, Mac. Robertson Land																	
FM-1	B	1260	660	19.80	±	1.16	2711	±	63	2158	±	136	4.04	±	0.2	simple	(7)
FM-2	B	1187	587	18.63	±	1.10	933	±	26	569	±	37	5.64	±	0.3	simple	(7)
BG-1	B	1200	600	18.84	±	1.11	2392	±	57	1905	±	121		—			(7)
BG-2	E	803	203	13.38	±	0.79	1015	±	38	933	±	65		—			(7)
BG-3	B	782	182	13.13	±	0.77	954	±	29	884	±	59		—			(7)
BG-4-R	E	771	171	13.00	±	0.76	759	±	20	680	±	44	4.40	±	0.2	simple	(7)
BG-5	E	771	171	13.00	±	0.76	648	±	22	566	±	38		—			(7)
Deep Freeze Range, Victoria Land																	
K3	B	1090	840	17.47	±	1.03	2255	±	122	1956	±	156		—		(s)	(21)
B2	E	980	780	15.90	±	0.94	487	±	26	330	±	26		—		(s)	(21)
K5	E	560	310	10.90	±	0.64	40	±	3	37	±	4		—			(21)
B3	E	950	750	15.49	±	0.91	19	±	2	12	±	1		—			(21)
ABL1	B	1022	722	16.09	±	0.95	2040	±	190	1900	±	209	5.00	±	0.9	simple	(8)

ABB1	E	596	296	11.23 ± 0.66	158 ± 12	145 ± 14	—	(c)	(8)
ABB2	E	596	296	11.12 ± 0.65	145 ± 14	134 ± 15	—	(c)	(8)
ABB3	E	608	308	10.08 ± 0.59	159 ± 12	164 ± 16	—	(c)	(8)
ABB4	E	520	220	9.29 ± 0.55	129 ± 10	144 ± 14	—	(c)	(8)
ABB6	E	500	200	9.95 ± 0.59	498 ± 41	569 ± 58	5.82 ± 0.9	simple	(8)
ABB7	E	500	200	9.95 ± 0.59	479 ± 29	544 ± 46	6.28 ± 0.9	simple	(8)
ABB8	E	430	130	9.60 ± 0.56	140 ± 9	151 ± 13	6.39 ± 1.1	simple	(8)
ABB10	E	393	93	8.98 ± 0.53	381 ± 23	472 ± 40	4.70 ± 0.7	complex	(8)
BROW1	E	470	170	9.57 ± 0.56	315 ± 21	357 ± 32	—	(c)	(8)
BROW2	E	474	174	9.91 ± 0.58	87 ± 7	90 ± 9	—	(c)	(8)
BROW3	E	505	205	10.10 ± 0.59	546 ± 45	621 ± 63	—	(c)	(8)
BROW7	E	674	374	11.82 ± 0.70	156 ± 12	136 ± 13	7.05 ± 1.3	simple	(8)
BROW8	E	660	360	11.67 ± 0.69	146 ± 14	129 ± 14	—	(c)	(8)
BROW10	E	284	0	8.24 ± 0.48	20 ± 5	24 ± 6	4.95 ± 2.3	complex	(8)
BROW11	E	290	0	8.20 ± 0.48	8 ± 1	10 ± 1	4.63 ± 1.6	complex	(8)

* We used a sample density of 2.7 g cm^{-3}, an attenuation length of 160 g cm^{-2}.

Supplementary Table 4. Modern and Pliocene atmospheric pressure by altitude

	Present			Pliocene (+3˚C)		
h (m)	500	1000	2000	500	1000	2000
T (˚C)	-13.46	-16.71	-23.21	-10.46	-13.71	-20.21
P (hPa)	926.5	867.2	757.7	927.2	868.5	760.0

Supplementary Note 1

Area 1 Study area and samples

1. Sør Rondane Mountains, Droning Maud Land

The Sør Rondane Mountains form the eastern portion of a discontinuous, ~2,000 km east-west trending range that is located 200 – 400 km inland[16] (Supplementary Figure 1). Geomorphologic evidence suggest that the ice sheet at this location was 400 m thicker than present[16], and cosmogenic nuclide concentrations indicate this area was first exposed at ~ 3 Ma[12]. Glacial tills were interpreted to reflect five distinct exposure stages, the oldest beginning prior to 4 Ma and the youngest from ~10 ka to present[16].

Samples were collected in the central and eastern parts of Sør Rondane Mountains during JARE-27, 28, 31 and 32. Supplementary Table 1 shows latitude, longitude, and altitude together with the production rate at each sampling sites. Sample 90111606 was collected from the highest point of Balchenfjella, in the eastern part of the mountains, and Sample 90111605 was collected near site of 90111606. Sample 012202 is an erratic retrieved from the flat top of a small nunatak located at Bard Glacier (Byrdbreen). Sample 012501 was collected from atop of a small nunatak located downstream of Bard Glacier. Sample 011401 was collected from the top of local peak near the Bergersenfjella. Samples 010404 and 010807 were taken from the top of the northern ridge of Mefjell, within the central portion of the mountains. Sample 870121-02 was collected from the top of roche moutennee located Walnumfjella, also within the central portion of the mountains. Sample 870121-01 was acquired from a position 100 m below the top of the roche moutennee.

2. Grove Mountains, Princess Elizabeth Land

Princess Elizabeth Land is located between 73°E and 87°E in East Antarctica. The Grove Mountains are located in the inland portion of Princess Elizabeth Land and consists of 64 nunataks that are scattered across a ~3200 km^2 area[17] (Supplementary Figure 2). Presently, most mountainous areas are covered by the EAIS to an altitude of ~1900 – 2000 m (ref. 2). Striations and moraines are observed at an altitude below ~100

m from the present ice surface[9]. The ages reported by previous studies[2,9-11] are from a vertical transect of bedrock samples collected from Mount Harding (R9201 to R9216). Huang *et al*. (ref. 2) and Lilly *et al*. (ref. 10) also reported ages from the Zakharoff Ridge (R8201 to R8210) and surrounding, unnamed nunataks (GR41 to GR25).

Supplementary Note 2

Area 2 Study area and samples

1. Ricker Hills, Victoria Land

Victoria Land is located between 164°E, 70°S and 166°E, 78°S in East Antarctica. This region fronts the Ross Sea and Ross Ice Shelf, contains abundant ice-free areas, and includes McMurdo Sound–Dry Valleys and the Transantarctic. This area is home to a large number of research bases and has therefore been well studied, with a number of ice sheet reconstructions reported.

The Ricker Hills are located about 100 km inland from the Ross Sea coast in southern Victoria Land[3] (Supplementary Figure 3). The ice-free areas are located in the David Glacier system on the edge of the East Antarctic Ice Sheet. The upper part of glacial trimline of these areas has been strongly weathered. At least five glacial drifts of differing deposition ages exist in these areas[18]. Strasky *et al*. (ref. 3) measured cosmogenic ^{10}Be and ^{21}Ne from seven erratic boulders.

2. Allan Hills, Victoria Land

The Allan Hills are located in southern Victoria Land (Supplementary Figure 4). Nishiizumi *et al*. (ref. 12) measured cosmogenic ^{10}Be and ^{26}Al in rock samples from the Allan Hills. Tschudi *et al*. (ref. 13) reported exposure ages from boulders of the Sirius Formation of the Allan Hills and bedrock directly underlying the Sirius Formation.

3. McMurdo Sound–Dry Valleys, Victoria Land

The McMurdo Sound–Dry Valleys are a series of glacial valleys in southern Victoria Land. There are three primary, large, moraine-covered valleys, the Victoria Valley, the Wright Valley, and the Taylor Valley (Supplementary Figure 5). Brown *et al*. (ref. 14) and Brook *et al*. (ref. 15) reported exposure ages of boulders from the Arena Valley, and Brook *et al*. (ref. 5) reported ^{10}Be and ^{26}Al data from boulders in glacial deposits of the Arena Valley and Mount Fleming.

4. Shackleton Range, Coats Land

Coats Land is located between 20°W and 36°W in East Antarctica. The Shackleton Range is located several tens of kilometers inland from the Filchner Ice Shelf[6] (Supplementary Figure 6). The outlet glaciers from the EAIS flow to the north and south of the range. Relatively less-weathered moraines are located at an altitude below ~340 m on Mount Provender[20]. Fogwill *et al*. (ref. 6) reported three exposure ages from the Shackleton Range, Coats Land. Two bedrock samples (SB1/J29 and SB2/J31) were acquired from the horizontal bedrock surface of plateau, and one bedrock sample (SB2/J31) was obtained from the summit of an ice-molded hill[6]. As for the sampling sites of plateau locations, glacial topography is present at one site, while this other is more weathered[6].

Supplementary Note 3

Area 3 Study area and samples

1. Prince Charles Mountains, Mac Robertson Land

Mac Robertson Land is located between 60°E and 70°E in East Antarctica. The Prince Charles Mountains form a ~400 km east-west trending range and are located 300 – 700 km inland (Supplementary Figure 7). Fink *et al*. (ref. 7) presented a deglacial chronology based on cosmogenic ^{10}Be and ^{26}Al exposure ages of the northern Prince Charles Mountains. They reported seven exposure ages from two areas; Fisher Massif (FM-1 and FM-2) and Battye Glacier (BG-1 to BG-5) (Supplementary Figure 7).

2. Deep Freeze Range, Victoria Land

The Deep Freeze Range is located within the Transantarctic Mountains in northern Victoria Land. Two glaciers (Campbell glacier and Priestley glacier) exist in the Deep Freeze Range with local mountain glaciers occupying the area in between[21] (Supplementary Figure 8). Oberholzer *et al*. (ref. 21) and Nicola *et al*. (ref. 8) reported exposure ages from several nunataks of the Deep Freeze Range (Supplementary Figure 8). They obtained exposure ages and erosion rates of glacially rounded bedrock and erratics.

Supplementary Note 4

Modern and Pliocene atmospheric pressure

The temperature and atmospheric pressure by altitude are expressed as.

$$\mathrm{T} = T_0 - 0.0065(h - h_0) \quad (1)$$

$$\mathrm{P} = P_S(1 - \frac{0.0065h}{T + 0.0065h + 273.15})^{5.257} \quad (2)$$

Where T and T_0 are temperature (˚C) of the target and reference points, respectively, and h and h_0 are altitude (m) of the target and reference points, respectively. P and P_s are atmospheric pressure (hPa) at the target point and sea level, respectively. Syowa Station is used as a reference point (altitude: 29.18 m; annual mean temperature: -10.4 ˚C), and sea-level pressure is 989.1 hPa (ref. 23). The calculated temperature and atmospheric

pressure at each altitude for the present and Pliocene (assuming a 3˚C warming) are shown in Supplementary Table 4.

Supplementary References

1. Moriwaki, K. & Hirakawa, K. Glacial Landforms and Late Cenozoic History of The Western Sør-Rondane Mountains. *Antarc. Rec.* **36**, 15–48 (1992). (in Japanese with English abstract)
2. Huang, F. *et al.* Fluctuation history of the interior East Antarctic Ice Sheet since mid-Pliocene. *Antarc. Sci.* **20**, 197–203 (2008).
3. Strasky, S. *et al.* Surface exposure ages imply multiple low-amplitude Pleistocene variations in East Antarctic Ice Sheet, Ricker Hills, Victoria Land. *Antarc. Sci.* **21**, 59–69 (2009).
4. Ross, P. –S. *et al.* Geological evolution of the Coombs-Allan Hills area, Ferrar large igneous province, Antarctica: Debris avalanches, mafic pyroclastic density currents, phreatocauldrons. *J. Volcanol. Geotherm. Res.* **172**, 38–60 (2008).
5. Brook, E. J. *et al.* Constraints on age, erosion, and uplift of Neogene glacial deposits in the Transantarctic Mountains determined from in situ cosmogenic ^{10}Be and ^{26}Al. *Geology* **23**, 1063–1066 (1995).
6. Fogwill, C. J. *et al.* Cosmogenic nuclides ^{10}Be and ^{26}Al imply limited Antarctic Ice Sheet thickening and low erosion in the Shackleton Range for > 1 m.y.. *Geology* **32**, 265–268 (2004).
7. Fink, D. *et al.* Pleistocene deglaciation chronology of the Amery Oasis and Radok Lake, northern Prince Charles Mountains, Antarctica. *Earth Planet. Sci. Lett.* **243**, 229–243 (2006).
8. Nicola, L. D. *et al.* Multiple cosmogenic nuclides document complex Pleistocene exposure history of glacial drifts in Terra Nova Bay (northern Victoria Land, Antarctica). *Quat. Res.* **71**, 83–92 (2009).

9. Kong, P. *et al.* Late Miocene ice sheet elevation in the Grove Mountains, East Antarctica, inferred from cosmogenic ^{21}Ne-^{10}Be-^{26}Al. *Glob. Planet. Chang.* **72**, 50–54 (2010).
10. Lilly, K. *et al.* Pleistocene dynamics of the interior East Antarctic ice sheet. *Geology* **38**, 703–706 (2010).
11. Liu, X. *et al.* History of ice sheet elevation in East Antarctica: Paleoclimatic implications. *Earth Planet. Sci. Lett.* **290**, 281–288 (2010).
12. Nishiizumi, K. *et al.* Cosmic ray produced ^{10}Be and ^{26}Al in Antarctic rocks: Exposure and erosion history. *Earth Planet. Sci. Lett.* **104**, 440–454 (1991).
13. Tschudi, S. *et al.* Surface exposure dating of Sirius Formation at Allan Hills nunatak, Antarctica: New evidence for long-term ice-sheet stability. *Eclogae Geol. Helv.* **96**, 109–114 (2003).
14. Brown, E. T. *et al.* Examination of surface exposure ages of Antarctic moraines using *in situ* produced ^{10}Be and ^{26}Al. *Geochim. Cosmochim. Acta* **55**, 2269–2283 (1991).
15. Brook, E. J. *et al.* Chronology of Taylor Glacier Advances in Arena Valley, Antarctica, Using *in situ* Cosmogenic ^{3}He and ^{10}Be. *Quat. Res.* **39**, 11–23 (1993).
16. Moriwaki, K., Hirakawa, K. & Matsuoka, N. Weathering stage of till and glacial history of the central Sør Rondane Mountains, East Antarctica. *Proc. NIPR Symp. Antarc. Geosci.* **5**, 99–111 (1991).
17. Liu, X. H. *et al.* Geology of the Grove Mountains in East Antarctica: new evidence for the final suture of Gondwana Land. *Sci. China* **46**, 305–319 (2003).
18. Baroni, C. *et al.* The Ricker Hills tillite provides evidence of Oligocene warm-based glaciation in Victoria Land, Antarctica. *Glob. Planet. Chang.* **60**, 457–470 (2008).
19. Brook, E. J. *et al.* Chronology of Taylor Glacier Advances in Arena Valley, Antarctica, Using *in situ* Cosmogenic ^{3}He and ^{10}Be. *Quat. Res.* **39**, 11–23 (1993).
20. Höfle, H. -C. and Buggisch, W. Glacial geology and petrography of erratics in the Shackleton Range, Antarctica. *Polarforschung* **63**, 183–201 (1995).
21. Oberholzer, P. *et al.* Limited Pliocene/Pleistocene glaciations in Deep Freeze Range, northern Victoria Land, Antarctica, derived from *in situ* cosmogenic

nuclides. *Antarc. Sci.* **15**, 493–502 (2003).

22. Matsuoka, N. *et al.* Quaternary bedrock erosion and landscape evolution in the Sør Rondane Mountains, East Antarctica: Reevaluating rates and processes. *Geomorphology* **81**, 408–420 (2006).

23. Stone, J. O. Air pressure and cosmogenic isotope production. *J. Geophys. Res.* **105**, 23753–23759 (2000).
